# A two-phase procedure for non-normal quantitative trait genetic association study

**DOI:** 10.1186/s12859-016-0888-x

**Published:** 2016-01-28

**Authors:** Wei Zhang, Huiyun Li, Zhaohai Li, Qizhai Li

**Affiliations:** Key Laboratory of Systems Control, Academy of Mathematics and Systems Science, Chinese Academy of Sciences, Beijing, 100190 China; School of Management and Economics, Beijing Institute of Technology, Beijing, 100081 China; Department of Statistics, George Washington University, Washington, 20052 DC USA

**Keywords:** Model selection, Quantitative trait genetic association studies, Robustness, Two-phase procedure

## Abstract

**Background:**

The nonparametric trend test (NPT) is well suitable for identifying the genetic variants associated with quantitative traits when the trait values do not satisfy the normal distribution assumption. If the genetic model, defined according to the mode of inheritance, is known, the NPT derived under the given genetic model is optimal. However, in practice, the genetic model is often unknown beforehand. The NPT derived from an uncorrected model might result in loss of power. When the underlying genetic model is unknown, a robust test is preferred to maintain satisfactory power.

**Results:**

We propose a two-phase procedure to handle the uncertainty of the genetic model for non-normal quantitative trait genetic association study. First, a model selection procedure is employed to help choose the genetic model. Then the optimal test derived under the selected model is constructed to test for possible association. To control the type I error rate, we derive the joint distribution of the test statistics developed in the two phases and obtain the proper size.

**Conclusions:**

The proposed method is more robust than existing methods through the simulation results and application to gene DNAH9 from the Genetic Analysis Workshop 16 for associated with Anti-cyclic citrullinated peptide antibody further demonstrate its performance.

**Electronic supplementary material:**

The online version of this article (doi:10.1186/s12859-016-0888-x) contains supplementary material, which is available to authorized users.

## Background

The past decades have witnessed many biological and epidemiological discoveries through the experimental design of genetic association studies based on the development of biological technology. Many variants have been identified to be associated with the quantitative traits. For example, in studying genetic loci in association with various phenotypes, 180 were reported to be associated with human height [1], 106 were associated with age at menarche [2], 97 were identified to be associated with body mass index [3], and the single-nucleotide polymorphism (SNP) rs4702 was associated with both diastolic and systolic blood pressure levels [4]. A standard approach to conduct an association test in a quantitative trait genetic study is to fit a linear model based on the assumption that the original or transformed trait values follow a normal distribution. However, the normal assumption is often violated for many traits even though some transformations such as the Log-transformation are carried out. For example, the number of tumors per subject in mouse follows a negative binomial distribution [5] and the survival time of a person follows a truncated distribution [6]. A good alternative to address this issue is to use the nonparametric tests.

Although there are various nonparametric tests in the literature, the most commonly used ones in genetic studies are the Kruskal-Wallis test (denote it by KW) [7] and the Jonckheere-Tepstra test (denote it by JT) [8, 9]. Originally, the KW was designed to detect the differences of the response variable in the medians of three groups and it was a nonparametric version of one-way analysis of variance based on ranking. The JT was also a rank-based test for an ordered alternative hypothesis which was particularly sensitive to the genetic mode of inheritance. Recently, Zhang and Li [10] defined the nonparametric risk and nonparametric odds and proposed a nonparametric trend test (NPT) that has been shown to be more powerful than KW and JT under a given genetic model. These methods, however, would suffer from loss of power when the underlying genetic model is misspecified.

In the present paper, we propose a two-phase robust procedure to test the genetic-phenotypic association. We first construct a test to classify the genetic model in a nonparametric way. We find that the test statistic tends to be positive when the genetic model is dominant, and negative when the model is recessive. Then based on the chosen model, the association test is conducted. We derive the correlation coefficient of the test used for choosing the genetic model and that for doing association study and obtain the proper size for a given nominal significance level. Extensive simulation studies are conducted to show the new approach to have empirical size less than the nominal level, and to compare this new approach with KW and MAX3, the maximum value of three NPTs. The results show that the proposed two-phase procedure is more robust than MAX3 and KW in the sense that its minimum power in a set of plausible models is the highest among the tests under consideration. Finally, a real data analysis is used for further illustration.

## Methods

### Notations and genetic models

Consider a biallelic marker whose genotype is coded as 0,1, and 2, corresponding to the count of a certain candidate risk allele or a minor allele. Suppose that there are *n* subjects that are independently sampled from a source population in a quantitative trait genetic association study. Let (*y*_*i*_,*g*_*i*_),*i*=1,2,⋯,*n* be the observed sample, where *y*_*i*_ is the trait value and *g*_*i*_ denotes the genotype value of the *i*th subject, *i*=1,2,⋯,*n*. For brevity, let the first *n*_0_ subjects have genotype 0, the second *n*_1_ subjects have genotype 1, and the last *n*_2_ subjects possess genotype 2. Denote *f*_*ij*_=Pr(*Y*_*i*_<*Y*_*j*_),*i*,*j*=0,1,2, where *Y*_0_,*Y*_1_ and *Y*_2_ are the random variables that take values in three sets $\{y_{1},y_{2},\cdots,y_{n_{0\phantom {\dot {i}\!}}}\}$, $\{y_{n_{0}+1},y_{n_{0}+2},\cdots,y_{n_{0}+n_{1}} \}$ and $\{y_{n_{0}+n_{1}+1}, y_{n_{0}+n_{1}+2}, \cdots, y_{n}\},$ respectively. The null hypothesis of no association is given by *H*_0_:*f*_01_=*f*_02_=1/2. The alternative hypothesis is *H*_1_:*f*_02_≥*f*_01_≥1/2 and *f*_02_>1/2.

A genetic model specifies the mode of inheritance. The three genetic models are: recessive model (REC) if *f*_01_=1/2 and *f*_12_=*f*_02_>1/2, additive model (ADD) if *f*_01_=*f*_12_>1/2 and *f*_02_>1/2, and dominant model (DOM) if $f_{01}=f_{02} > \frac {1}{2}$ and *f*_12_=1/2.

### Model selection

**Fig. 1:**
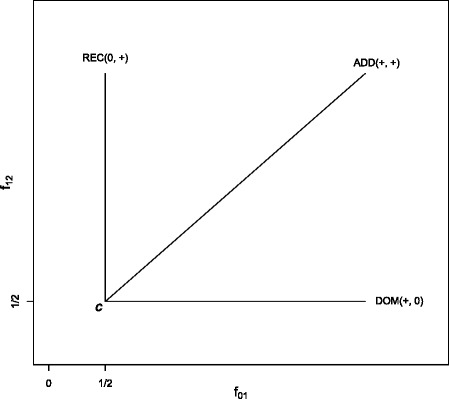
The common three genetic models in the genetic model space. The point *C*=(1/2,1/2) corresponds to the null hypothesis

Denote *Δ*_1_=*f*_01_−1/2, *Δ*_2_=*f*_12_−1/2. We find that *Δ*_1_−*Δ*_2_ tends to be negative value under the recessive model and take positive under the dominant model. The signs of (*Δ*_1_,*Δ*_2_) under the three genetic models are plotted in Fig. 1, where the line corresponding to the additive model is the straight line with a slope of 1 at the point *C*, *C*=(1/2,1/2)^*τ*^ and *τ* denotes the transpose of a vector or a matrix, and the other two lines are for the recessive and dominant models, respectively. The recessive and dominant models form the boundaries of the space under the alternative hypothesis. The vertex *C* corresponds to the null hypothesis. Denote

$$\begin{aligned} {}\hat{f}_{01}&=\frac{1}{n_{0}n_{1}}\sum\limits_{i=1}^{n_{0}}\sum\limits_{j=n_{0}+1}^{n_{0}+n_{1}}I(y_{i}<y_{j}),\\ {}\hat{f}_{12}&=\frac{1}{n_{1}n_{2}}\sum\limits_{j=n_{0}+1}^{n_{0}+n_{1}}\sum\limits_{k=n_{0}+n_{1}+1}^{n}I(y_{j}<y_{k}),\\ {}\widehat\sigma_{01}^{2}&=\frac{n_{1}-1}{{n_{0}^{2}}n_{1}}\sum\limits_{i=1}^{n_{0}}\left[\frac{1}{n_{1}} \sum\limits_{j=n_{0}+1}^{n_{0}+n_{1}}I(y_{i}<y_{j})-1/2\right]^{2}\\ {}&\quad+\!\frac{n_{0}\,-\,1}{n_{0}{n_{1}^{2}}}\sum\limits_{j=n_{0}+1}^{n_{0}+n_{1}}\left[\frac{1}{n_{0}}\!\sum\limits_{i=1}^{n_{0}}I(y_{i}\!<\!y_{j})\,-\,1/2\right]^{2} \,+\,\frac{1}{4n_{0}n_{1}},\\ {}\widehat\sigma_{01,12}^{2}&=\frac{1}{{n_{1}^{2}}}\sum\limits_{j=n_{0}+1}^{n_{0}+n_{1}}\left[\frac{1}{n_{0}}\sum\limits_{i=1}^{n_{0}} I(y_{i}<y_{j})-1/2\right]\\ &\quad\left[\frac{1}{n_{2}}\sum\limits_{k=n_{0}+n_{1}+1}^{n}I(y_{j}<y_{k})-1/2\right], \end{aligned} $$

and

$$\begin{aligned} {} \widehat\sigma_{12}^{2}&=\frac{n_{2}-1}{{n_{1}^{2}}n_{2}}\sum\limits_{j=n_{0}+1}^{n_{0}+n_{1}} \left[\frac{1}{n_{2}}\sum\limits_{k=n_{0}+n_{1}+1}^{n}I(y_{i}<y_{k})-1/2\right]^{2}\\ &\quad+\frac{n_{1}-1}{n_{1}{n_{2}^{2}}}\sum\limits_{k=n_{0}+n_{1}+1}^{n}\left[\frac{1}{n_{1}}\sum\limits_{j=n_{0}+1}^{n_{0}+n_{1}}I(y_{j}<y_{k})-1/2\right]^{2}\\ &\quad+\displaystyle\frac{1}{4n_{1}n_{2}}. \end{aligned} $$

Then $\hat f_{01}$ and $\hat f_{12}$ are the consistent estimators of *f*_01_ and *f*_12_, respectively, $\widehat \sigma _{01}^{2}$ and $\widehat \sigma _{12}^{2}$ are, respectively, the consistent estimators of the variances of $\hat f_{01}$ and $\hat f_{12}$, and $\widehat \sigma _{01,12}^{2}$ is the consistent estimator of the covariance between $\hat f_{01}$ and $\hat f_{12}$. Define a test statistic for genetic model selection as

$$\begin{array}{*{20}l} Z_{1}=\frac{\hat f_{01}-\hat f_{12}}{\sqrt{\hat\sigma_{01}^{2}-2\hat\sigma_{01,12}^{2}+\hat\sigma_{12}^{2}}}. \end{array} $$

Under the null hypothesis, *Z*_1_ asymptotically follows the standard normal distribution. So the genetic models can be determined as follows: i) if *Z*_1_>*ξ* (>0), then the genetic model is dominant; ii) if *Z*_1_<−*ξ*, then the genetic model is recessive; otherwise, the additive model is claimed. Here, *ξ* is set to be the 90 % quantile of the standard normal distribution.

### The nonparametric test under a given genetic model

Denote

$$\begin{aligned} {}\hat{f}_{02}&=\frac{1}{n_{0}n_{2}}\sum\limits_{i=1}^{n_{0}}\sum\limits_{k=n_{0}+n_{1}+1}^{n}I(y_{i}<y_{k}),\\ {}\hat{f}_{R}&=\frac{1}{(n_{0}+n_{1})n_{2}}\sum\limits_{i=1}^{n_{0}+n_{1}}\sum_{k=n_{0}+n_{1}+1}^{n}I\{y_{i}<y_{k}\}\\ &=\frac{n_{0}}{n_{0}+n_{1}}\hat f_{02} + \frac{n_{1}}{n_{0}+n_{1}}\hat f_{12},\\ {}\hat\sigma_{02}^{2}&=\frac{n_{2}-1}{{n_{0}^{2}}n_{2}}\sum\limits_{i=1}^{n_{0}}\left[\frac{1}{n_{2}}\sum\limits_{k=n_{0}+n_{1}+1}^{n}I(y_{i}<y_{k})-1/2\right]^{2}\\ &\quad\!\,+\,\frac{n_{0}\,-\,1}{n_{0}{n_{2}^{2}}}\sum\limits_{k=n_{0}+n_{1}+1}^{n}\!\left[\!\frac{1}{n_{0}}\sum\limits_{i=1}^{n_{0}}I\!(y_{i}\!\!<\!\!y_{k})\,-\,\!1/2\!\right]^{2}\!\,+\,\!\frac{1}{4n_{0}n_{2}},\\ {}\widehat\sigma_{02,12}^{2}&=\frac{1}{{n_{2}^{2}}}\sum\limits_{k=n_{0}+n_{1}+1}^{n}\left[\frac{1}{n_{0}}\sum\limits_{i=1}^{n_{0}}I(y_{i}<y_{k})-1/2\right]\\ &\quad\left[\frac{1}{n_{1}}\sum\limits_{j=n_{0}+1}^{n_{0}+n_{1}}I(y_{j}<y_{k})-1/2\right], \end{aligned} $$

and

$${}{\widehat\sigma_{R}^{2}} =\frac{{n_{0}^{2}}}{(n_{0}+n_{1})^{2}}\widehat{\sigma}_{02}^{2} + \frac{2n_{0}n_{1}}{(n_{0}+n_{1})^{2}}\widehat{\sigma}_{02,12}^{2} + \frac{{n_{1}^{2}}}{(n_{0}+n_{1})^{2}}\widehat{\sigma}_{12}^{2}. $$

Then the NPT under the recessive model can be given by $Z_{R} = (\hat f_{R}-1/2)/\hat \sigma _{R}$.

Let

$$\begin{aligned} {}\hat{f}_{01}&\,=\,\frac{1}{n_{0}n_{1}}\sum\limits_{i=1}^{n_{0}}\sum\limits_{j=n_{0}+1}^{n_{0}+n_{1}}I(y_{i}<y_{j}),\\ {}{w}_{1}^{*}&\,=\,\sqrt{\!(n_{0}\,+\,n_{1})\!\big/\!\left[\!(n\,+\,n_{1}\!)\widehat \sigma_{01}^{2}\!\right]}, w_{2}^{*}\,=\,\sqrt{\!(n_{1}\,+\,n_{2})\!\big/\!\left[\!(n\,+\,n_{1}\!)\widehat \sigma_{12}^{2}\right]},\\ {}w_{1}&\,=\,\frac{w_{1}^{*}}{w_{1}^{*}+w_{2}^{*}}, w_{2}=\frac{w_{2}^{*}}{w_{1}^{*}+w_{2}^{*}} \end{aligned} $$

and

$$\begin{array}{*{20}l} {}{\hat{f}}_{A}=w_{1}\hat f_{01}+w_{2}\hat f_{12}, {\widehat\sigma^{2}_{A}}= {w_{1}^{2}}\widehat\sigma_{01}^{2} + 2w_{1}w_{2}\widehat\sigma_{01,12}^{2} + {w_{2}^{2}}\widehat\sigma_{12}^{2}. \end{array} $$

Then, the NPT under the additive genetic model is $Z_{A} =(\hat f_{A}-1/2)/\hat \sigma _{A}$.

Similarly, denote

$${\fontsize{8.1pt}{9.6pt}{\begin{aligned} {}\widehat\sigma_{01}^{2}&=\frac{n_{1}-1}{{n_{0}^{2}}n_{1}}\sum\limits_{i=1}^{n_{0}}\left[\frac{1}{n_{1}}\sum\limits_{j=n_{0}+1}^{n_{0}+n_{1}} I(y_{i}<y_{j})-1/2\right]^{2}\\ &\quad+\!\frac{n_{0}\,-\,1}{n_{0}{n_{1}^{2}}}\sum\limits_{j=n_{0}+1}^{n_{0}+n_{1}}\left[\frac{1}{n_{0}}\sum\limits_{i=1}^{n_{0}}I(y_{i}\!<\!y_{j})\,-\,1/2\!\right]^{2} \,+\,\frac{1}{4n_{0}n_{1}},\\ {}\widehat{\sigma}_{01,02}^{2}&=\frac{1}{{n_{0}^{2}}}\sum\limits_{i=1}^{n_{0}}\left[\frac{1}{n_{1}}\sum\limits_{j=n_{0}+1}^{n_{0}+n_{1}} I(y_{i}<y_{j})-1/2 \right]\\ &\quad\left[\frac{1}{n_{2}}\sum\limits_{k=n_{0}+n_{1}+1}^{n}I(y_{i}<y_{k})-1/2\right].\\ {}\hat{f}_{D}&=\frac{1}{n_{0}(n_{1}+n_{2})}\sum\limits_{i=1}^{n_{0}}\sum_{j=n_{0}+1}^{n}I\{y_{i}\!<y_{j}\}=\frac{n_{1}}{n_{1}+n_{2}}\hat f_{01} + \frac{n_{2}}{n_{1}+n_{2}}\hat f_{02}, \end{aligned}}} $$

and

$$\begin{array}{*{20}l} {}{\widehat\sigma_{D}^{2}}=\frac{{n_{1}^{2}}}{(n_{1}+n_{2})^{2}}\widehat{\sigma}_{01}^{2} + \frac{2n_{1}n_{2}}{(n_{1}+n_{2})^{2}}\widehat{\sigma}_{01,02}^{2} + \frac{{n_{2}^{2}}}{(n_{1}+n_{2})^{2}}\widehat{\sigma}_{02}^{2}. \end{array} $$

Then the NPT under the dominant model is $Z_{D} = (\hat f_{D}-1/2)/\hat \sigma _{D}$. Under the null hypothesis, *Z*_*R*_, *Z*_*A*_ and *Z*_*D*_ follow the standard normal distribution.

### Two-phase procedure

We propose a two-phase procedure (TPP) for the quantitative trait association study by first determining the underlying genetic model in the first phase, followed by testing the association with the corresponding NPT for the selected model in the second phase. In details, the two-phase procedure can be described by the following two steps:

**Step 1.** Determine the genetic model using *Z*_1_. If *Z*_1_<−*ξ*, the recessive model is used, else if *Z*_1_>*ξ*, we use the dominant model, otherwise, the additive model is used.

**Step 2.** We choose the association test statistic based on the chosen model in Step 1 and do the association study.

### Size adjustment

To adjust the size of the two-phase procedure for a given overall nominal significance level, we need to derive the joint distribution of *Z*_1_ and *Z*_*x*_, *x*∈{*R*,*A*,*D*}. From the Additional file 1, under the null hypothesis, (*Z*_1_,*Z*_*x*_)^*τ*^ asymptotically follows a bivariate normal distribution with mean (0,0) and *Λ*_*x*_, where

$$\Lambda_{x}=\left(\begin{array}{cc} 1&\rho_{x}\\ \rho_{x}&1 \end{array} \right),~~~x\in\{R, A, D\}. $$

Denote the cumulative distribution function of *Y*_0_, *Y*_1_ and *Y*_2_ by *F*_0_, *F*_1_ and *F*_2_, respectively. Then *ρ*_*R*_, *ρ*_*A*_ and *ρ*_*D*_ are functions of *F*_0_,*F*_1_,*F*_2_ and *p* (the minor allele frequency, or MAF, for short), which can be estimated empirically based on the observed data. The consistent estimates can be obtained under the situation that the means of the trait values in the groups with different genotypes being equal. The technical details of derivations for *ρ*_*R*_, *ρ*_*A*_ and *ρ*_*D*_ under the null hypothesis are presented in the Additional file 1. Suppose that the null hypothesis is rejected at the level of *α*^∗^ in the second phase. Then, to control the overall level at a given *α*, we have $\alpha =\text {P}_{H_{0}}\left (Z_{1} < -\xi, |Z_{R}|>z(1-\alpha ^{*}/2)\right)+ \text {P}_{H_{0}}\left (|Z_{1}| < \xi, |Z_{A}|>z(1-\alpha ^{*}/2)\right) + \text {P}_{H_{0}}\left (Z_{1} > \xi, |Z_{D}|>\right. \left.z(1-\alpha ^{*}/2)\right)$, where *z*(*α*) is the *α* quantile of the standard normal distribution. So, this relation can be written as

$${\fontsize{8.1pt}{9.6pt}{\begin{aligned} {}\alpha&\,=\,\displaystyle\int_{\Omega_{R}}\left\{\Phi\left(\frac{\!-z(1\,-\,\alpha^{*}/2)\,-\,\rho_{R}u}{(1\,-\,{\rho_{R}^{2}})^{1/2}}\right)\,+\, \Phi\!\left(\frac{-z(1\,-\,\alpha^{*}/2)\,+\,\rho_{R}u}{(1\,-\,{\rho_{R}^{2}})^{1/2}}\right)\!\right\}\!\mathrm{d}\Phi(u) \\ {}&\quad+\displaystyle\int_{\Omega_{A}}\!\!\left\{\!\Phi\!\left(\!\frac{-z(1\,-\,\alpha^{*}/2)\,-\,\rho_{A}u}{(1\,-\,{\rho_{A}^{2}})^{1/2}}\!\right)\,+\, \Phi\!\left(\!\frac{-z(1\,-\,\alpha^{*}\!/2)\,+\,\rho_{A}u}{(1\,-\,{\rho_{A}^{2}})^{1/2}}\!\right)\!\right\}\!\mathrm{d}\Phi(u)\\ {}&\quad+\!\! \displaystyle \int_{\Omega_{D}}\!\left\{\!\Phi\!\left(\frac{\!-z(1\,-\,\alpha^{*}\!/2)\,-\,\rho_{D}u}{(1\,-\,{\rho_{D}^{2}})^{1/2}}\!\right)\!+ \!\Phi\!\left(\frac{\!-z(1\,-\,\alpha^{*}\!/2)\,+\,\rho_{D}u}{(1\,-\,{\rho_{D}^{2}})^{1/2}}\!\right)\!\right\}\!\mathrm{d}\Phi(u), \end{aligned}}} $$

where *Ω*_*R*_={*u*:*u*<−*ξ*}, *Ω*_*A*_={*u*:−*ξ*≤*u*≤*ξ*}, *Ω*_*D*_={*u*:*u*>*ξ*}, and *Φ*(·) is the cumulative distribution function of the standard normal distribution. Under the null hypothesis, we can numerically calculate the adjusted significant level for the association test statistic in the second phase. Table 2 shows the mean and standard error of *α*^∗^ with the nominal level of 0.05 and 0.001 based on 1,000 and 50,000 replicates, respectively. It indicates that *α*^∗^ is more likely to be smaller than *α*, and also *α*^∗^ is less vulnerable to the MAF. For example, when MAF=0.25, the adjusted levels for the nominal *α*=0.05 and *α*=0.001 are 0.0360 and 0.00065, and the corresponding standard error are 0.0003 and 0.000013, respectively.

## Results

### The performance of model selection procedure

We conduct simulation studies to explore the performance of the model selection procedure. We generate data considering three genetic models. Consider the linear model *Y*=*β*_0_+*Gβ*_1_+*ε*, where *Y* denotes the phenotype value, *G* denotes the genotype value at a SNP locus, and *ε* follows a truncated generalized extreme value distribution (a heavy-tailed distribution, denoted as tGEV(0, 0, *d*, 0)) with the shape parameter 0, the location parameter 0, the scale parameter *d*, and the truncated point 0. Here we specify *β*_0_=0.50, *β*_1_=0.50, *d*=5, and the MAF *p*∈{0.05,0.10,⋯,0.50}. The total sample size is 1,500. 10,000 replicates are conducted to compute the true selection rate (TSR) under different scenarios. Table 1 shows the results for *ξ*=*Φ*^−1^(0.90)=1.282. The other results for *ξ*=*Φ*^−1^(0.80)=0.841, *ξ*=*Φ*^−1^(0.85)=1.036 and *Φ*^−1^(0.95)=1.645 are given in the Additional file 1. From Table 1, we can see that the TSR increases as MAF increases. For example, if the recessive model is true, the TSR is 19.48 % for MAF of 0.05, while it is 86.21 % for MAF of 0.50. It makes sense since the expected number of subjects with genotype 2 is increasing with the MAF increasing. We also find that the TSR for additive model is satisfactory with the TSR being around 80 %. For example, the TSR are 79.23 % and 80.09 % corresponding to MAF of 0.05 and 0.50, respectively. Besides this, we also conduct simulations with covariates considering *Y*=*β*_0_+*Xγ*+*Gβ*_1_+*ε*, where *X* is a covariate. The detailed results are available in the Additional file 1.

**Table 1:** The true selecting rate (*%*) of genetic model using *Z*_1_ with *ξ*=*Φ* ^−1^(0.9) when the error follows tGEV(0,0,5,1)

True model	REC				ADD				DOM			
MAF ∖Selection rate		REC	ADD	DOM		REC	ADD	DOM		REC	ADD	DOM
0.05		19.48	75.59	4.93		8.21	79.23	12.56		2.40	73.34	24.26
0.10		34.80	63.67	1.53		8.85	80.21	10.94		1.37	64.52	34.11
0.15		50.25	49.30	0.45		8.96	81.14	9.90		0.59	52.69	46.72
0.20		61.19	38.60	0.21		9.63	80.22	10.15		0.27	39.68	60.05
0.25		71.12	28.84	0.04		9.44	80.69	9.87		0.08	30.44	69.48
0.30		77.44	22.54	0.02		9.62	80.33	10.05		0.05	22.96	76.99
0.35		81.94	18.04	0.02		10.00	80.37	9.63		0.04	18.00	81.96
0.40		84.64	15.34	0.02		9.56	80.45	9.99		0.02	15.00	84.98
0.45		85.69	14.30	0.01		9.85	80.33	9.82		0.00	13.91	86.09
0.50		86.21	13.75	0.04		10.16	80.09	9.75		0.02	14.14	85.84

The sample size is *n*=1,500 and 10,000 replicates are conducted

### The adjusted significant level

Table 2 shows the adjusted *α*^∗^ of the TPP under the null hypothesis. The parameter setting is the same as above. When the nominal level is 0.05, we calculate the mean and standard deviation (SD) based on 2,000 replicates. And 50,000 replicates are conducted for the nominal level of 0.001. The results indicate that the adjusted level is always less than the nominal significant level *α*. For example, when MAF =0.25, the adjusted levels for the nominal level *α*=0.05 and *α*=0.001 are 0.0310 and 0.00059, respectively. And the value of *α*^∗^ is relatively stable because its standard deviation can be omitted compared with the means. For example, when MAF =0.1, the adjusted levels for the nominal level *α*=0.05 and *α*=0.001 are 0.0335 and 0.00063, and the corresponding standard deviations are 0.00169 and 0.000039, respectively.

**Table 2:** The adjusted level *α* ^∗^ for the nominal significant level *α* of 0.05 and 0.001

	MAF	0.05	0.10	0.15	0.20	0.25	0.30	0.35	0.40	0.45	0.50
*α*=0.05	mean	0.0364	0.0335	0.0327	0.0318	0.0310	0.0303	0.0297	0.0293	0.0290	0.0290
	sd	0.00689	0.00169	0.00173	0.00148	0.00120	0.00093	0.00074	0.00062	0.00058	0.00057
*α*=0.001	mean	0.00071	0.00063	0.00063	0.00061	0.00059	0.00058	0.00057	0.00056	0.00056	0.00056
	sd	0.000151	0.000039	0.000034	0.000032	0.000024	0.000016	0.000010	0.000005	0.000003	0.000002

1,000 replicates are for the nominal level 0.05 and 50,000 replicates are for the level 0.001

### Type I error rate

We evaluate the empirical type I error rates of five tests: KW, *Z*_*R*_, *Z*_*A*_, MAX3, and TPP. The simulation settings are similar as above. The sample size is 1,500. Here we use *ξ*=*Φ*^−1^(0.90), *β*_0_=0.50, and *p*∈{0.05,0.10,⋯,0.50}. 2,000 replicates are conducted for the nominal significant level of 0.05 and 50,000 replicates are conducted for the nominal significant level of 0.001. Table 3 shows the empirical type I errors of the five tests under the significant level of 0.05 and 0.001. The results show that all of the five tests could control the type I error correctly with the empirical values being close to the nominal significance level. For example, when MAF =0.20, the empirical type I error rates of KW, *Z*_*R*_, *Z*_*A*_, MAX3, and TPP test are 0.046, 0.048, 0.051, 0.045, and 0.041, respectively, under the significant level of 0.05. When MAF =0.35 and the nominal significant level is 0.001, the empirical type I error rates of KW, *Z*_*R*_, *Z*_*A*_, MAX3, and TPP test are 0.00090, 0.00086, 0.00098, 0.00090, and 0.00080, respectively.

**Table 3:** The empirical type I errors of KW, *Z*_*R*_, *Z*_*A*_, MAX3, and TPP when the error term follows tGEV(0,0,5,0)

	*α*=0.05						*α*=0.001				
MAF	KW	*Z*_*R*_	*Z*_*A*_	MAX3	TPP		KW	*Z*_*R*_	*Z*_*A*_	MAX3	TPP
0.05	0.049	0.031	0.057	0.032	0.043		0.00064	0.00035	0.00114	0.00078	0.00082
0.10	0.051	0.055	0.047	0.045	0.047		0.00076	0.00040	0.00092	0.00062	0.00060
0.15	0.049	0.055	0.051	0.057	0.050		0.00098	0.00062	0.00092	0.00070	0.00080
0.20	0.046	0.048	0.051	0.045	0.041		0.00098	0.00088	0.00094	0.00102	0.00092
0.25	0.058	0.049	0.052	0.058	0.050		0.00090	0.00074	0.00076	0.00088	0.00068
0.30	0.057	0.054	0.049	0.058	0.044		0.00112	0.00084	0.00088	0.00114	0.00086
0.35	0.056	0.052	0.051	0.055	0.047		0.00090	0.00086	0.00098	0.00090	0.00080
0.40	0.052	0.048	0.045	0.050	0.038		0.00114	0.00100	0.00082	0.00106	0.00070
0.45	0.048	0.049	0.054	0.057	0.043		0.00090	0.00086	0.00090	0.00108	0.00074
0.50	0.052	0.050	0.044	0.044	0.034		0.00078	0.00080	0.00068	0.00090	0.00064

The sample size is 1,500. The left panel is for the significant level *α*=0.05 and the right panel is for the significant level *α*=0.001

### Power

**Fig. 2:**
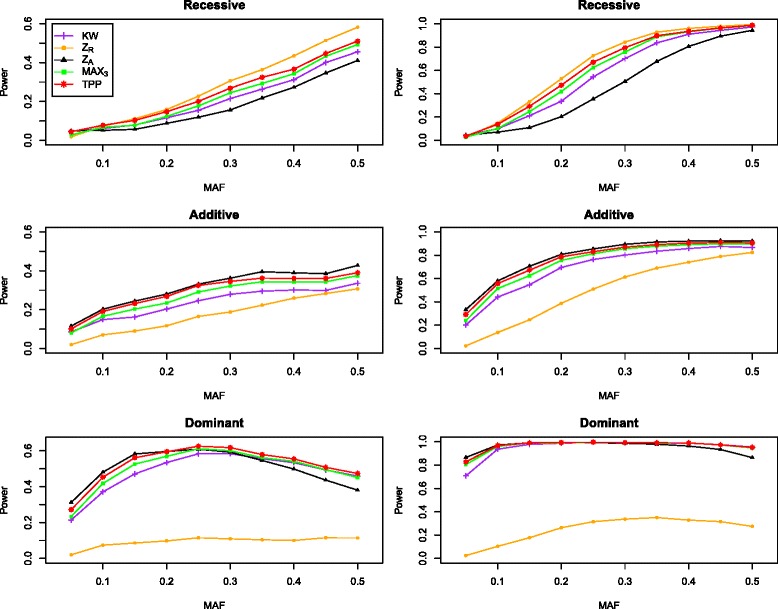
The powers of KW, *Z*_*R*_, *Z*_*A*_, MAX3, and TPP with tGEV(0,0,5,0) error under three genetic models. The nominal level is 0.05. The first column is for *β*_1_=0.25 and the second column is for *β*_1_=0.50. The total number of the subjects is *n*=1,500

**Fig. 3:**
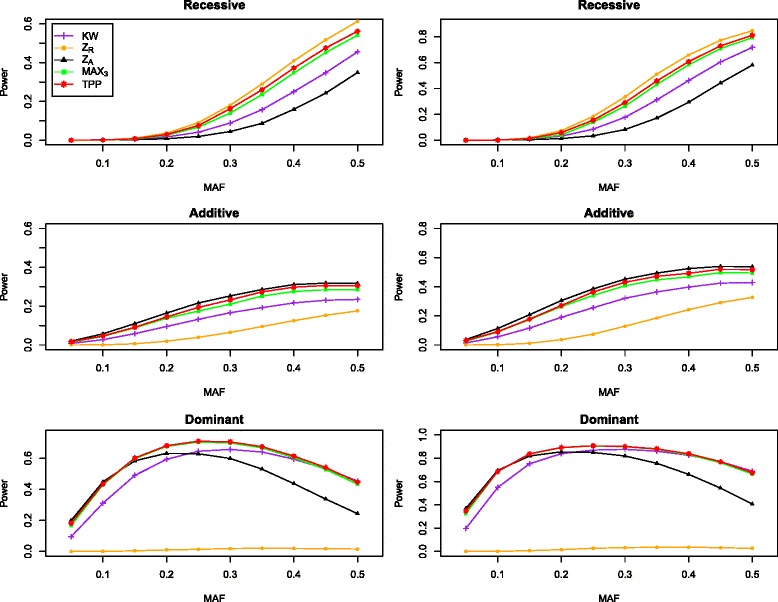
The powers of KW, *Z*_*R*_, *Z*_*A*_, MAX3, and TPP with tGEV(0,0,*d*,0) error under three genetic models. The nominal level is 0.001. The first column is for *β*_1_=0.25,*d*=3 and the second column is for *β*_1_=0.50,*d*=5. The total number of the subjects is *n*=1,500

We compare the power among KW, *Z*_*R*_, *Z*_*A*_, MAX3 and TPP under the similar settings to those described above. Figures 2 and 3 report the power results for the nominal level of 0.05 and 0.001, respectively, under the recessive, additive, and dominant models. In order to make the power comparable, when the nominal level is 0.001, we specify *d*=3 for *β*_1_=0.25 and *d*=5 for *β*_1_=0.50, and when the nominal level is 0.05, we set *d*=5 and *β*_1_={0.25,0.50}. The results indicate that, except the NPT test under the true genetic model, the proposed TPP is always more powerful than KW and MAX3. this makes sense because that NPT under a given model (*Z*_*R*_, *Z*_*A*_) is the most powerful under that model, and the model selection procedure always gives a large probability of TSR. TPP is more powerful than KW, *Z*_*A*_, and MAX3 under the recessive model and in most scenarios under the dominant model. In some cases, there are 6 *%* power increase. For example, when MAF is 0.20, *β*_1_=0.50, *α*=0.05 and the genetic model is recessive, the empirical powers of KW, *Z*_*A*_, MAX3, and TPP are 0.335, 0.202, 0.418, and 0.473, respectively. The performance of TPP is superior than the other three test KW, *Z*_*R*_ and MAX3 when the true model is additive or dominant. For example, when MAF is 0.30 and the genetic model is additive, *β*_1_=0.50, *α*=0.001, the empirical powers of KW, *Z*_*R*_, MAX3, and TPP are 0.321, 0.128, 0.407, and 0.431, respectively. Furthermore, using *Z*_*R*_ under the additive or dominant model can result in substantial loss of power. The TPP has higher robustness against the genetic model than other four tests. For example, when *α*=0.05 and *β*_1_=0.50, the minimum value of power for TPP over MAF from 0.10 to 0.50 is 0.137 under the recessive, additive and dominant model, which is larger than those of KW (0.099), *Z*_*R*_ (0.103), *Z*_*A*_ (0.070), and MAX3 (0.112).

### Application to gene DNAH9 associated with anti-CCP measure

We apply KW, *Z*_*A*_, MAX3 and TPP to identify the deleterious SNPs in the gene DNAH9 [11] for the association with the anti-CCP measure using the data from Genetic Workshop 16 [12, 13]. The anti-CCP is present in the blood of the majority of patients with rheumatoid arthritis (RA). The data includes 867 cases (with anti-CCP) and 1,195 controls (without anti-CCP). Here we impute them with the minimum value of the anti-CCP values in cases, which is 20.053 following Zheng et al. (2012)[14]. We remove the effect of population stratification using four principal coordinates [15] following Zhang and Li [10] and take the residuals as the new outcome. There are 92 SNPs in gene DNAH9 on Chromosome 17. We calculate the p-values of these SNPs using the KW, *Z*_*A*_, MAX3 and TPP approaches. There are six SNPs in gene DNAH9 whose proportions of the missing genotype value are more than 15 *%*, so we only show the p-value of the remaining 86 SNPs. In the main text, we shows the results of the SNPs whose *p*-values are relatively small (almost less than 0.05) in Table 4 and the *p*-values of the other SNPs are summarized in Table S10 in the Additional file 1. We find that the SNP rs11655963 has the minimum *p*-value of 2.72×10^−5^ using the TPP. The corresponding *p*-values using KW, *Z*_*A*_, and MAX3 are 1.18×10^−4^, 8.40×10^−5^ and 7.44×10^−5^, respectively. Burton et al.(2007)[16] proposed to use the p-value threshold of 5×10^−5^ as the moderate association at the genome-wide level. Because the *p*-values of KW, *Z*_*A*_ and MAX are all larger than 5×10^−5^, there are no moderate genome-wide associations. However, for the TPP, we calculate the adjusted p-value threshold for 5×10^−5^ and it is 3.64×10^−5^. This indicates that using the TPP can give the moderate-strong effect.

**Table 4:** The *p*-values of 17 SNPs in gene *DNAH9* for the association with Anti-CCP Measure

snpid	KW	*Z*_*A*_	MAX3	TPP	Genetic model	*α* ^∗^
rs9896319	0.0024	0.0542	0.0060	0.0031	REC	3.16×10^−5^
rs736626	0.1008	0.0337	0.0720	0.0337	ADD	2.98×10^−5^
rs4791473	0.0621	0.0214	0.0436	0.0214	ADD	2.99×10^−5^
rs12946617	0.1182	0.0459	0.0962	0.0459	ADD	3.75×10^−5^
rs7223160	0.0894	0.0289	0.0624	0.0289	ADD	2.98×10^−5^
rs7207282	0.1039	0.0345	0.0738	0.0345	ADD	2.98×10^−5^
rs11657375	0.1359	0.0412	0.0880	0.0412	ADD	3.29×10^−5^
rs11651010	0.0002	0.0001	0.0001	0.0001	ADD	3.03×10^−5^
rs3744580	0.0804	0.0390	0.0561	0.0390	ADD	2.99×10^−5^
rs11655963	1.18×10^−4^	8.40×10^−5^	7.44×10^−5^	2.72×10^−5^	REC	3.64×10^−5^
rs12936861	0.0529	0.0594	0.0336	0.0146	DOM	2.87×10^−5^
rs9896309	0.0356	0.1863	0.0446	0.0205	DOM	2.86×10^−5^
rs7215021	0.1275	0.0384	0.0715	0.0384	ADD	3.48×10^−5^
rs9303041	0.0507	0.1855	0.0327	0.0149	DOM	3.16×10^−5^
rs10445247	0.0481	0.0320	0.0491	0.0320	ADD	2.92×10^−5^
rs3764845	0.0719	0.0325	0.0698	0.0325	ADD	2.91×10^−5^
rs1990236	0.0622	0.0217	0.0365	0.0217	ADD	3.27×10^−5^

*α* ^∗^ is the adjusted p-value threshold for 5×10^−5^. The sixth column (denoted by Genetic model) is the selected genetic model using the TPP in the first phase

## Discussion and Conclusion

With the developments of biological technology, more and more data on quantitative traits and genotypes are generated and deposited in public database such as The National Center for Biotechnology Information database. It is urgent to develop new methods to excavate useful information to help understand the etiology of human complex diseases. A nonparametric two-phase procedure is proposed here to test the association between a di-allelic SNP and a non-normal distributed quantitative trait when the genetic model is unknown. Simulation results show that the proposed TPP is more robust than the existing methods.

If there are covariates needed to be adjusted for, we can first regress on the covariates and use the residuals as the new outcome and then employ TPP to conduct the association study. The detailed simulation results are presented in Additional file 1. Besides the truncated generalized extreme value distributional (a heavy-tailed distribution) error term with the truncation point 0, we also consider the error term following the centralized *t* distribution and general generalized extreme value distribution, respectively. The results are given in Additional file 1, where the similar results are observed.

## Additional file

Additional file 1
**The derivations of** ***ρ***_***R***_ **,** ***ρ***_***A***_ **and** ***ρ***_***D***_ **under the null hypothesis.** Consistent estimators of *ρ*_*R*_, *ρ*_*A*_ and *ρ*_*D*_ under the null hypothesis. Additional simulation results for the model selection procedure. Simulation results for the error term following the generalized extreme distribution. Simulation results for the error term following the centralized t distribution. Simulation results for the model with covariates. Additional p-value results of the SNPs in gene DNAH9 for the associated with Anti-CCP Measure. (PDF 179 kb)
