# Supplementary material for: A two-phase procedure for non-normal quantitative trait genetic association study
Source: BMC Bioinformatics. 2016 Jan 28;17:52. doi: 10.1186/s12859-016-0888-x (PMC4730615; doi:10.1186/s12859-016-0888-x)
Supplement: Additional file 1 — The derivations of ρ R , ρ A and ρ D under the null hypothesis. Consistent estimators of ρ R, ρ A and ρ D under the null hypothesis. Additional simulation results for the model selection procedure. Simulation results for the error term following the generalized extreme distribution. Simulation results for the error term following the centralized t distribution. Simulation results for the model with covariates. Additional p-value results of the SNPs in gene DNAH9 for the associated with Anti-CCP Measure. (PDF 179 kb) [file 12859_2016_888_MOESM1_ESM.pdf]

An additional file for

A two-phase procedure for non-normal quantitative trait genetic association study

Wei Zhang, Huiyun Li, Zhaohai Li and Qizhai Li

Contents

1. Derivations of ρ_R, ρ_A and ρ_D under the null hypothesis

2. Consistent estimators of ρ_R, ρ_A and ρ_D under the null hypothesis

3. Additional simulation results for the model selection procedure

4. Simulation results for the error term following the generalized extreme distribution

5. Simulation results for the error term following the centralized t distribution

6. Simulation results for the model with covariates

7. Additional p-values results of the SNPs in gene *DNAH9* for the association with Anti-CCP Measure

1. Derivations of ρ_R, ρ_A and ρ_D under the null hypothesis

The covariance of Z_1 and Z_x under the null hypothesis can be expressed as

$$\mathrm{cov}_{H_0}\big(Z_1, Z_x\big) = \frac{\mathrm{cov}_{H_0}\big(\hat{f}_{01} - \hat{f}_{12}, \hat{f}_x - 1/2\big)}{\sqrt{\mathrm{var}_{H_0}\big(\hat{f}_{01} - \hat{f}_{12}\big)\mathrm{var}_{H_0}\big(\hat{f}_x - 1/2\big)}}, \quad x \in \{R, A, D\}.$$

Using the notations in the main text, we have, under the null hypothesis,

$$\mathrm{var}_{H_0}\big(\hat{f}_{01}-1/2\big) = \frac{1}{n_0 n_1}\Big\{(n_1-1)E_{H_0}\big[1/2 - F_1(Y_0)\big]^2 + (n_0-1)E_{H_0}\big[F_0(Y_1) - 1/2\big]^2 + 1/4\Big\},$$

$$\mathrm{var}_{H_0}\big(\hat{f}_{12}-1/2\big) = \frac{1}{n_1 n_2}\Big\{(n_2-1)E_{H_0}\big[1/2 - F_2(Y_1)\big]^2 + (n_1-1)E_{H_0}\big[F_1(Y_2) - 1/2\big]^2 + 1/4\Big\},$$

and

$$\text{cov}_{H_0}\big(\hat{f}_{01}-1/2,\hat{f}_{12}-1/2\big)=\frac{1}{n_1}E_{H_0}\big[F_0(Y_1)-1/2\big]\big[1/2-F_2(Y_1)\big].$$

So

$$\text{var}_{H_0}\big(\hat{f}_{01}-\hat{f}_{12}\big)=\text{var}_{H_0}\big(\hat{f}_{01}-1/2\big)-2\text{cov}_{H_0}\big(\hat{f}_{01}-1/2,\hat{f}_{12}-1/2\big)+\text{var}_{H_0}\big(\hat{f}_{12}-1/2\big).$$

And we have

$$\begin{aligned}
&\text{var}_{H_0}\big(\hat{f}_R-1/2\big)\\
&=\frac{n_0}{n_2(n_0+n_1)^2}\Big\{(n_2-1)E_{H_0}\big[1/2-F_2(Y_0)\big]^2+(n_0-1)E_{H_0}\big[F_0(Y_2)-1/2\big]^2+1/4\Big\}\\
&\quad+\frac{n_1}{n_2(n_0+n_1)^2}\Big\{(n_2-1)E_{H_0}\big[1/2-F_2(Y_1)\big]^2+(n_1-1)E_{H_0}\big[F_1(Y_2)-1/2\big]^2+1/4\Big\}\\
&\quad+\frac{2n_0n_1}{n_2(n_0+n_1)^2}E_{H_0}\big[F_0(Y_2)-1/2\big]\big[F_1(Y_2)-1/2\big].
\end{aligned}$$

Define $\lambda_1^*=\sqrt{(n_0+n_1)/\big[(n+n_1)\sigma_{01}^2\big]}$, $\lambda_2^*=\sqrt{(n_1+n_2)/\big[(n+n_1)\sigma_{12}^2\big]}$, $\lambda_1=\frac{\lambda_1^*}{\lambda_1^*+\lambda_2^*}$, $\lambda_2=\frac{\lambda_2^*}{\lambda_1^*+\lambda_2^*}$, then asymptotically, we have

$$\begin{aligned}
&\text{var}_{H_0}\big(\hat{f}_A-1/2\big)\\
&=\lambda_1^2\text{var}_{H_0}\big(\hat{f}_{01}-1/2\big)+2\lambda_1\lambda_2\text{cov}_{H_0}\big(\hat{f}_{01}-1/2,\hat{f}_{12}-1/2\big)+\lambda_2^2\text{var}_{H_0}\big(\hat{f}_{12}-1/2\big)\\
&=\frac{\lambda_1^2}{n_0n_1}\Big\{(n_1-1)E_{H_0}\big[1/2-F_1(Y_0)\big]^2+(n_0-1)E_{H_0}\big[F_0(Y_1)-1/2\big]^2+1/4\Big\}\\
&\quad+\frac{\lambda_2^2}{n_1n_2}\Big\{(n_2-1)E_{H_0}\big[1/2-F_2(Y_1)\big]^2+(n_1-1)E_{H_0}\big[F_1(Y_2)-1/2\big]^2+1/4\Big\}\\
&\quad+\frac{2\lambda_1\lambda_2}{n_1}E_{H_0}\big[F_0(Y_1)-1/2\big]\big[1/2-F_2(Y_1)\big],
\end{aligned}$$

and

$$\begin{aligned}
&\text{var}_{H_0}\big(\hat{f}_D-1/2\big)\\
&=\frac{n_1}{n_0(n_1+n_2)^2}\Big\{(n_1-1)E_{H_0}\big[1/2-F_1(Y_0)\big]^2+(n_0-1)E_{H_0}\big[F_0(Y_1)-1/2\big]^2+1/4\Big\}\\
&\quad+\frac{n_2}{n_0(n_1+n_2)^2}\Big\{(n_2-1)E_{H_0}\big[1/2-F_2(Y_0)\big]^2+(n_0-1)E_{H_0}\big[F_0(Y_2)-1/2\big]^2+1/4\Big\}\\
&\quad+\frac{2n_1n_2}{n_0(n_1+n_2)^2}E_{H_0}\big[1/2-F_1(Y_0)\big]\big[1/2-F_2(Y_0)\big].
\end{aligned}$$

Next we derive the covariance of the $\hat{f}_{01}-\hat{f}_{12}$ and $\hat{f}_x-1/2$, $x\in\{R,A,D\}$ under the null hypothesis,

$$\text{cov}_{H_0}\big(\hat{f}_{01}-\hat{f}_{12},\hat{f}_R-1/2\big)$$

$$
\begin{aligned}
&= \mathrm{cov}_{H_0}\left(\hat{f}_{01}-\hat{f}_{12}, \frac{n_0}{n_0+n_1}\hat{f}_{02}+\frac{n_1}{n_0+n_1}\hat{f}_{12}-1/2\right)\\
&= \frac{n_0}{n_0+n_1}\mathrm{cov}_{H_0}\big(\hat{f}_{01}-1/2, \hat{f}_{02}-1/2\big)+\frac{n_1}{n_0+n_1}\mathrm{cov}_{H_0}\big(\hat{f}_{01}-1/2, \hat{f}_{12}-1/2\big)\\
&\quad -\frac{n_0}{n_0+n_1}\mathrm{cov}_{H_0}\big(\hat{f}_{12}-1/2, \hat{f}_{02}-1/2\big)-\frac{n_1}{n_0+n_1}\mathrm{cov}_{H_0}\big(\hat{f}_{12}-1/2, \hat{f}_{12}-1/2\big)\\
&= \frac{1}{n_0+n_1}E_{H_0}\big[1/2-F_1(Y_0)\big]\big[1/2-F_2(Y_0)\big]+\frac{1}{n_0+n_1}E_{H_0}\big[F_0(Y_1)-1/2\big]\big[1/2-F_2(Y_1)\big]\\
&\quad -\frac{n_0}{n_2(n_0+n_1)}E_{H_0}\big[F_0(Y_2)-1/2\big]\big[F_1(Y_2)-1/2\big]-\frac{n_2-1}{n_2(n_0+n_1)}E_{H_0}\big[1/2-F_2(Y_1)\big]^2\\
&\quad -\frac{n_1-1}{n_2(n_0+n_1)}E_{H_0}\big[F_1(Y_2)-1/2\big]^2-\frac{1}{4n_2(n_0+n_1)},
\end{aligned}
$$

and asymptotically,

$$
\begin{aligned}
&\mathrm{cov}_{H_0}\big(\hat{f}_{01}-\hat{f}_{12}, \hat{f}_A-1/2\big)\\
&= \lambda_1\mathrm{var}_{H_0}\big(\hat{f}_{01}-1/2\big)+(\lambda_2-\lambda_1)\mathrm{cov}_{H_0}\big(\hat{f}_{01}-1/2, \hat{f}_{12}-1/2\big)-\lambda_2\mathrm{var}_{H_0}\big(\hat{f}_{12}-1/2\big),
\end{aligned}
$$

and

$$
\begin{aligned}
&\mathrm{cov}_{H_0}\big(\hat{f}_{01}-\hat{f}_{12}, \hat{f}_D-1/2\big)\\
&= \mathrm{cov}_{H_0}\left(\hat{f}_{01}-\hat{f}_{12}, \frac{n_1}{n_1+n_2}\hat{f}_{01}+\frac{n_2}{n_1+n_2}\hat{f}_{02}-1/2\right)\\
&= \frac{n_1}{n_1+n_2}\mathrm{cov}_{H_0}\big(\hat{f}_{01}-1/2, \hat{f}_{01}-1/2\big)+\frac{n_2}{n_1+n_2}\mathrm{cov}_{H_0}\big(\hat{f}_{01}-1/2, \hat{f}_{02}-1/2\big)\\
&\quad -\frac{n_1}{n_1+n_2}\mathrm{cov}_{H_0}\big(\hat{f}_{12}-1/2, \hat{f}_{01}-1/2\big)-\frac{n_2}{n_1+n_2}\mathrm{cov}_{H_0}\big(\hat{f}_{12}-1/2, \hat{f}_{02}-1/2\big)\\
&= \frac{n_1-1}{n_0(n_1+n_2)}E_{H_0}\big[1/2-F_1(Y_0)\big]^2+\frac{n_0-1}{n_0(n_1+n_2)}E_{H_0}\big[F_0(Y_1)-1/2\big]^2+\frac{1}{4n_0(n_1+n_2)}\\
&\quad -\frac{1}{n_1+n_2}E_{H_0}\big[1/2-F_2(Y_1)\big]\big[F_0(Y_1)-1/2\big]-\frac{1}{n_1+n_2}E_{H_0}\big[F_1(Y_2)-1/2\big]\big[F_0(Y_2)-1/2\big]\\
&\quad +\frac{n_2}{n_0(n_1+n_2)}E_{H_0}\big[1/2-F_1(Y_0)\big]\big[1/2-F_2(Y_0)\big].
\end{aligned}
$$

Assume that $n_0/n \longrightarrow p_0, n_1/n \longrightarrow p_1$, and $n_2/n \longrightarrow p_2$ as $n \longrightarrow \infty$, where $p_0, p_1, p_2 \in (0,1)$. The variances of $\hat{f}_{01}$ and $\hat{f}_{12}$, and the covariance of $\hat{f}_{01}$ and $\hat{f}_{12}$ can be written by

$$
\begin{aligned}
n\mathrm{var}_{H_0}\big(\hat{f}_{01}-1/2\big) &= \frac{1}{p_0}E_{H_0}\big[1/2-F_1(Y_0)\big]^2+\frac{1}{p_1}E\big[F_1(Y_2)-1/2\big]+O(1/n),\\
n\mathrm{var}_{H_0}\big(\hat{f}_{12}-1/2\big) &= \frac{1}{p_1}E_{H_0}\big[1/2-F_2(Y_1)\big]^2+\frac{1}{p_2}E\big[F_1(Y_2)-1/2\big]^2+O(1/n),
\end{aligned}
$$

and

$$n\mathrm{cov}_{H_0}\big(\hat f_{01}-1/2,\hat f_{12}-1/2\big)=\frac{1}{p_1}E_{H_0}\big[F_0(Y_1)-1/2\big]\big[1/2-F_2(Y_1)\big].$$

Thus,

$$\begin{aligned}
&n\mathrm{var}_{H_0}\big(\hat f_{01}-\hat f_{12}\big)\\
&\quad=\frac{1}{p_0}E_{H_0}\big[1/2-F_1(Y_0)\big]^2+\frac{1}{p_1}E\big[F_1(Y_2)-1/2\big]-\frac{2}{p_1}E_{H_0}\big[F_0(Y_1)-1/2\big]\big[1/2-F_2(Y_1)\big]\\
&\quad\quad+\frac{1}{p_1}E_{H_0}\big[1/2-F_2(Y_1)\big]^2+\frac{1}{p_2}E\big[F_1(Y_2)-1/2\big]^2+O(1/n)\\
&\quad\triangleq V_{01,12}+O(1/n),
\end{aligned}$$

$$\begin{aligned}
&n\mathrm{var}_{H_0}\big(\hat f_R-1/2\big)\\
&\quad=\frac{p_0}{(p_0+p_1)^2}\left\{\frac{p_0}{p_2}E_{H_0}\big[F_0(Y_2)-1/2\big]^2+\frac{2p_1}{p_2}E_{H_0}\big[F_0(Y_2)-1/2\big]\big[F_1(Y_2)-1/2\big]\right\}\\
&\quad\quad+\frac{p_0}{(p_0+p_1)^2}E_{H_0}\big[1/2-F_2(Y_0)\big]^2+\frac{p_1}{(p_0+p_1)^2}E_{H_0}\big[1/2-F_2(Y_1)\big]^2\\
&\quad\quad+\frac{p_1^2}{p_2(p_0+p_1)^2}E_{H_0}\big[F_1(Y_2)-1/2\big]^2+O(1/n)\\
&\quad\triangleq\frac{V_R}{(p_0+p_1)^2}+O(1/n),
\end{aligned}$$

$$\begin{aligned}
&n\mathrm{var}_{H_0}\big(\hat f_A-1/2\big)\\
&\quad=\frac{\lambda_1^2}{p_0}E_{H_0}\big[1/2-F_1(Y_0)\big]^2+\frac{\lambda_1^2}{p_1}E\big[F_1(Y_2)-1/2\big]+\frac{2\lambda_1\lambda_2}{p_1}E_{H_0}\big[F_0(Y_1)-1/2\big]\big[1/2-F_2(Y_1)\big]\\
&\quad\quad+\frac{\lambda_2^2}{p_1}E_{H_0}\big[1/2-F_2(Y_1)\big]^2+\frac{\lambda_2^2}{p_2}E\big[F_1(Y_2)-1/2\big]^2+O(1/n)\\
&\quad\triangleq V_A+O(1/n),
\end{aligned}$$

and

$$\begin{aligned}
&n\mathrm{var}_{H_0}\big(\hat f_D-1/2\big)\\
&\quad=\frac{p_1}{(p_1+p_2)^2}\left\{\frac{p_1}{p_0}E_{H_0}\big[1/2-F_1(Y_0)\big]^2+\frac{2p_2}{p_0}E_{H_0}\big[1/2-F_1(Y_0)\big]\big[1/2-F_2(Y_0)\big]\right\}\\
&\quad\quad+\frac{p_1}{(p_1+p_2)^2}E_{H_0}\big[F_0(Y_1)-1/2\big]^2+\frac{p_2^2}{p_0(p_1+p_2)^2}E_{H_0}\big[1/2-F_2(Y_0)\big]^2\\
&\quad\quad+\frac{p_2}{(p_1+p_2)^2}E_{H_0}\big[F_0(Y_2)-1/2\big]^2+O(1/n)\\
&\quad\triangleq\frac{V_D}{(p_1+p_2)^2}+O(1/n).
\end{aligned}$$

The numerators of $\text{cov}_{H_0}\big(Z_1, Z_x\big), x \in \{R, A, D\}$ are

$$
\begin{aligned}
&n\text{cov}_{H_0}\big(\hat{f}_{01} - \hat{f}_{12}, \hat{f}_R - 1/2\big) \\
&\quad = \frac{1}{p_0+p_1}\Big\{E_{H_0}\Big[1/2 - F_1(Y_0)\Big]\Big[1/2 - F_2(Y_0)\Big] + E_{H_0}\Big[F_0(Y_1) - 1/2\Big]\Big[1/2 - F_2(Y_1)\Big]\Big\} \\
&\qquad - \frac{p_0}{p_2(p_0+p_1)}E_{H_0}\Big[F_0(Y_2) - 1/2\Big]\Big[F_1(Y_2) - 1/2\Big] - \frac{1}{p_0+p_1}E_{H_0}\Big[1/2 - F_2(Y_1)\Big]^2 \\
&\qquad - \frac{p_1}{p_2(p_0+p_1)}E_{H_0}\Big[F_1(Y_2) - 1/2\Big]^2 + O(1/n) \\
&\quad \triangleq -\frac{C_R}{p_0+p_1} + O(1/n),
\end{aligned}
$$

$$
\begin{aligned}
&n\text{cov}_{H_0}\big(\hat{f}_{01} - \hat{f}_{12}, \hat{f}_A - 1/2\big) \\
&\quad = \frac{\lambda_1}{p_0}E_{H_0}\big[1/2 - F_1(Y_0)\big]^2 + \frac{\lambda_1}{p_1}E\big[F_1(Y_2) - 1/2\big] + \frac{\lambda_2 - \lambda_1}{p_1}E_{H_0}\big[F_0(Y_1) - 1/2\big]\big[1/2 - F_2(Y_1)\big] \\
&\qquad - \frac{\lambda_2}{p_1}E_{H_0}\big[1/2 - F_2(Y_1)\big]^2 + \frac{\lambda_2}{p_2}E\big[F_1(Y_2) - 1/2\big]^2 + O(1/n) \\
&\quad \triangleq C_A + O(1/n),
\end{aligned}
$$

and

$$
\begin{aligned}
&n\text{cov}_{H_0}\big(\hat{f}_{01} - \hat{f}_{12}, \hat{f}_D - 1/2\big) \\
&\quad = -\frac{1}{p_1+p_2}\Big\{E_{H_0}\Big[1/2 - F_2(Y_1)\Big]\Big[F_0(Y_1) - 1/2\Big] + E_{H_0}\Big[F_1(Y_2) - 1/2\Big]\Big[F_0(Y_2) - 1/2\Big]\Big\} \\
&\qquad + \frac{p_2}{p_0(p_1+p_2)}E_{H_0}\Big[1/2 - F_1(Y_0)\Big]\Big[1/2 - F_2(Y_0)\Big] + \frac{1}{n(p_1+p_2)}E_{H_0}\Big[F_0(Y_1) - 1/2\Big]^2 \\
&\qquad + \frac{p_1}{p_0(p_1+p_2)}E_{H_0}\Big[1/2 - F_1(Y_0)\Big]^2 + O(1/n) \\
&\quad \triangleq \frac{C_D}{p_1+p_2} + O(1/n).
\end{aligned}
$$

Hence, asymptotically,

$$
\begin{aligned}
\text{corr}_{H_0}(Z_1, Z_R) &= -\frac{C_R}{\sqrt{V_{01,12}V_R}} + O(1/n), \\
\text{corr}_{H_0}(Z_1, Z_A) &= \frac{C_A}{\sqrt{V_{01,12}V_A}} + O(1/n), \\
\text{corr}_{H_0}(Z_1, Z_D) &= \frac{C_D}{\sqrt{V_{01,12}V_D}} + O(1/n),
\end{aligned}
$$

under the null hypothesis, $\rho_x = \text{corr}_{H_0}\big(Z_1, Z_x\big) = \text{cov}_{H_0}\big(Z_1, Z_x\big), x \in \{R, A, D\}$.

2. Consistent estimators of ρ_R, ρ_A and ρ_D under the null hypothesis

Because the distribution functions of Y_0, Y_1 and Y_2 are unknown, we need to estimate the distribution functions F_0, F_1 and F_2 in order to obtain the expressions of ρ_R, ρ_A and ρ_D under H_0. We point out that the direct empirical estimates of these correlations using the observed data are biased. So we modify the sample to make that the means of the three sets $\{y_1, y_2, \cdots, y_{n_0}\}$, $\{y_{n_0+1}, y_{n_0+2}, \cdots, y_{n_0+n_1}\}$, and $\{y_{n_0+n_1+1}, y_{n_0+n_1+2}, \cdots, y_n\}$ are equal. This adjustment will make sure the estimation procedure of ρ_R, ρ_A and ρ_D being calculated under the null hypothesis. First, we calculate the sample medians of the above three sets, denoted them by a_0, a_1 and a_2, respectively, where $a_0 = (\sum_{i=1}^{n_0} y_i)/n_0$, $a_1 = (\sum_{j=n_0+1}^{n_0+n_1} y_j)/n_1$ and $a_2 = (\sum_{k=n_0+n_1+1}^{n} y_k)/n_2$. Then we change the sample sets to $\{y_1 - a_0, y_2 - a_0, \cdots, y_{n_0} - a_0\}$, $\{y_{n_0+1} - a_1, y_{n_0+2} - a_1, \cdots, y_{n_0+n_1} - a_1\}$, $\{y_{n_0+n_1+1} - a_2, y_{n_0+n_1+2} - a_2, \cdots, y_n - a_2\}$, respectively. Denote the corresponding transformed samples by $\{\tilde{y}_1, \tilde{y}_2, \cdots, \tilde{y}_{n_0}\}$, $\{\tilde{y}_{n_0+1}, \tilde{y}_{n_0+2}, \cdots, \tilde{y}_{n_0+n_1}\}$ and $\{\tilde{y}_{n_0+n_1+1}, \tilde{y}_{n_0+n_1+2}, \cdots, \tilde{y}_n\}$. Then $\text{var}_{H_0}(\hat{f}_{01})$, $\text{var}_{H_0}(\hat{f}_{12})$ and $\text{cov}_{H_0}(\hat{f}_{01} - 1/2, \hat{f}_{12} - 1/2)$ under the null hypothesis can be estimated by $\widetilde{\sigma}^2_{01}, \widetilde{\sigma}^2_{12}$, and $\widetilde{\sigma}^2_{01,12}$, respectively, which results in $\widehat{\text{var}}_{H_0}(\hat{f}_{01} - \hat{f}_{12}) = \widetilde{\sigma}^2_{01} - 2\widetilde{\sigma}^2_{01,12} + \widetilde{\sigma}^2_{12}$, where $\widetilde{\sigma}^2_{01}, \widetilde{\sigma}^2_{12}$, and $\widetilde{\sigma}^2_{01,12}$ are given by

$$\widetilde{\sigma}^2_{01} = \frac{n_1 - 1}{n_0^2 n_1} \sum_{i=1}^{n_0} \Big[\frac{1}{n_1} \sum_{j=n_0+1}^{n_0+n_1} I(\tilde{y}_i < \tilde{y}_j) - 1/2\Big]^2 + \frac{n_0 - 1}{n_0 n_1^2} \sum_{j=n_0+1}^{n_0+n_1} \Big[\frac{1}{n_0} \sum_{i=1}^{n_0} I(\tilde{y}_i < \tilde{y}_j) - 1/2\Big]^2 + \frac{1}{4n_0 n_1},$$

$$\begin{aligned}\widetilde{\sigma}^2_{12} = &\frac{n_2 - 1}{n_1^2 n_2} \sum_{j=n_0+1}^{n_0+n_1} \Big[\frac{1}{n_2} \sum_{k=n_0+n_1+1}^{n} I(\tilde{y}_i < \tilde{y}_k) - 1/2\Big]^2 + \frac{1}{4n_1 n_2} \\ &+ \frac{n_1 - 1}{n_1 n_2^2} \sum_{k=n_0+n_1+1}^{n} \Big[\frac{1}{n_1} \sum_{j=n_0+1}^{n_0+n_1} I(\tilde{y}_j < \tilde{y}_k) - 1/2\Big]^2,\end{aligned}$$

and

$$\widetilde{\sigma}^2_{01,12} = \frac{1}{n_1^2} \sum_{j=n_0+1}^{n_0+n_1} \Big[\frac{1}{n_0} \sum_{i=1}^{n_0} I(\tilde{y}_i < \tilde{y}_j) - 1/2\Big]\Big[\frac{1}{n_2} \sum_{k=n_0+n_1+1}^{n} I(\tilde{y}_j < \tilde{y}_k) - 1/2\Big].$$

The estimates of the variance $\hat{f}_R, \hat{f}_A$ and $\hat{f}_D$ are given by

$$\widehat{\text{var}}_{H_0}\big(\hat{f}_R - 1/2\big) = \frac{n_0^2}{(n_0+n_1)^2}\widetilde{\sigma}_{02}^2 + \frac{2n_0n_1}{(n_0+n_1)^2}\widetilde{\sigma}_{02,12}^2 + \frac{n_1^2}{(n_0+n_1)^2}\widetilde{\sigma}_{12}^2,$$
$$\widehat{\text{var}}_{H_0}\big(\hat{f}_A - 1/2\big) = \tilde{w}_1^2\widetilde{\sigma}_{01}^2 + 2\tilde{w}_1\tilde{w}_2\widetilde{\sigma}_{01,12}^2 + \tilde{w}_2^2\widetilde{\sigma}_{12}^2,$$

and

$$\widehat{\text{var}}_{H_0}\big(\hat{f}_D - 1/2\big) = \frac{n_1^2}{(n_1+n_2)^2}\widetilde{\sigma}_{01}^2 + \frac{2n_1n_2}{(n_1+n_2)^2}\widetilde{\sigma}_{01,02}^2 + \frac{n_2^2}{(n_1+n_2)^2}\widetilde{\sigma}_{02}^2,$$

where $\tilde{w}_1^* = \sqrt{(n_0+n_1)/[(n+n_1)\widetilde{\sigma}_{01}^2]}$, $\tilde{w}_2^* = \sqrt{(n_1+n_2)/\big[(n+n_1)\widetilde{\sigma}_{12}^2\big]}$, $\tilde{w}_1 = \tilde{w}_1^*/(\tilde{w}_1^* + \tilde{w}_2^*)$, $\tilde{w}_2 = \tilde{w}_2^*/(\tilde{w}_1^* + \tilde{w}_2^*)$,

$$\widetilde{\sigma}_{02}^2 = \frac{n_2-1}{n_0^2n_2}\sum_{i=1}^{n_0}\Big[\frac{1}{n_2}\sum_{k=n_0+n_1+1}^{n} I(\tilde{y}_i < \tilde{y}_k) - 1/2\Big]^2 + \frac{1}{4n_0n_2}$$
$$+\frac{n_0-1}{n_0n_2^2}\sum_{k=n_0+n_1+1}^{n}\Big[\frac{1}{n_0}\sum_{i=1}^{n_0} I(\tilde{y}_i < \tilde{y}_k) - 1/2\Big]^2,$$
$$\widetilde{\sigma}_{02,12}^2 = \frac{1}{n_2^2}\sum_{k=n_0+n_1+1}^{n}\Big[\frac{1}{n_0}\sum_{i=1}^{n_0} I(\tilde{y}_i < \tilde{y}_k) - 1/2\Big]\Big[\frac{1}{n_1}\sum_{j=n_0+1}^{n_0+n_1} I(\tilde{y}_j < \tilde{y}_k) - 1/2\Big],$$

and

$$\widetilde{\sigma}_{01,02}^2 = \frac{1}{n_0^2}\sum_{i=1}^{n_0}\Big[\frac{1}{n_1}\sum_{j=n_0+1}^{n_0+n_1} I(\tilde{y}_i < \tilde{y}_j) - 1/2\Big]\Big[\frac{1}{n_2}\sum_{k=n_0+n_1+1}^{n} I(\tilde{y}_i < \tilde{y}_k) - 1/2\Big].$$

Next we will give the estimate of $\text{cov}_{H_0}(\hat{f}_{01} - \hat{f}_{12}, \hat{f}_x - 1/2)$, $x \in \{R, A, D\}$ under the null hypothesis. Because the expression of $\text{cov}_{H_0}(\hat{f}_{01} - \hat{f}_{12}, \hat{f}_R - 1/2)$ is

$$\begin{aligned}
&\text{cov}_{H_0}\big(\hat{f}_{01} - \hat{f}_{12}, \hat{f}_R - 1/2\big)\\
&= \frac{1}{n_0+n_1}E_{H_0}\big[1/2 - F_1(Y_0)\big]\big[1/2 - F_2(Y_0)\big] + \frac{1}{n_0+n_1}E_{H_0}\big[F_0(Y_1) - 1/2\big]\big[1/2 - F_2(Y_1)\big]\\
&\quad - \frac{n_0}{n_2(n_0+n_1)}E_{H_0}\big[F_0(Y_2) - 1/2\big]\big[F_1(Y_2) - 1/2\big] - \frac{n_2-1}{n_2(n_0+n_1)}E_{H_0}\big[1/2 - F_2(Y_1)\big]^2\\
&\quad - \frac{n_1-1}{n_2(n_0+n_1)}E_{H_0}\big[F_1(Y_2) - 1/2\big]^2 - \frac{1}{4n_2(n_0+n_1)},
\end{aligned}$$

the estimate of $\text{cov}_{H_0}\big(\hat{f}_{01} - \hat{f}_{12}, \hat{f}_R - 1/2\big)$ can be written as

$$\begin{aligned}
&\widehat{\text{cov}}_{H_0}\big(\hat{f}_{01} - \hat{f}_{12}, \hat{f}_R - 1/2\big)\\
&= \frac{1}{n_0(n_0+n_1)}\sum_{i=1}^{n_0}\Big[\frac{1}{n_1}\sum_{j=n_0+1}^{n_0+n_1} I(\tilde{y}_i < \tilde{y}_j) - 1/2\Big]\Big[\frac{1}{n_2}\sum_{k=n_0+n_1+1}^{n} I(\tilde{y}_i < \tilde{y}_k) - 1/2\Big]\\
&\quad + \frac{1}{n_1(n_0+n_1)}\sum_{j=n_0+1}^{n_0+n_1}\Big[\frac{1}{n_0}\sum_{i=1}^{n_0} I(\tilde{y}_i < \tilde{y}_j) - 1/2\Big]\Big[\frac{1}{n_2}\sum_{k=n_0+n_1+1}^{n} I(\tilde{y}_j < \tilde{y}_k) - 1/2\Big]
\end{aligned}$$

$$-\frac{n_0}{n_2^2(n_0+n_1)}\sum_{k=n_0+n_1+1}^{n}\Big[\frac{1}{n_0}\sum_{i=1}^{n_0}I(\tilde{y}_i<\tilde{y}_k)-1/2\Big]\Big[\frac{1}{n_1}\sum_{j=n_0+1}^{n_0+n_1}I(\tilde{y}_j<\tilde{y}_k)-1/2\Big]$$
$$-\frac{n_1}{n_0+n_1}\tilde{\sigma}_{12}^2.$$

Asymptotically, the expression of $\mathrm{cov}_{H_0}\Big(\hat{f}_{01}-\hat{f}_{12},\hat{f}_A-1/2\Big)$ is

$$\mathrm{cov}_{H_0}\big(\hat{f}_{01}-\hat{f}_{12},\hat{f}_A-1/2\big)$$
$$=\tilde{w}_1\mathrm{var}_{H_0}\big(\hat{f}_{01}-1/2\big)+(\tilde{w}_2-\tilde{w}_1)\mathrm{cov}_{H_0}\big(\hat{f}_{01}-1/2,\hat{f}_{12}-1/2\big)-\tilde{w}_2\mathrm{var}_{H_0}\big(\hat{f}_{12}-1/2\big).$$

So, $\mathrm{cov}_{H_0}\big(\hat{f}_{01}-\hat{f}_{12},\hat{f}_A-1/2\big)$ can be estimated by

$$\widehat{\mathrm{cov}}_{H_0}\big(\hat{f}_{01}-\hat{f}_{12},\hat{f}_A-1/2\big)=\tilde{w}_1\tilde{\sigma}_{01}^2+(\tilde{w}_2-\tilde{w}_1)\tilde{\sigma}_{01,12}^2-\tilde{w}_2\tilde{\sigma}_{12}^2.$$

Similarly, we have the expression of $\mathrm{cov}_{H_0}\big(\hat{f}_{01}-\hat{f}_{12},\hat{f}_D-1/2\big)$ as

$$\mathrm{cov}_{H_0}\big(\hat{f}_{01}-\hat{f}_{12},\hat{f}_D-1/2\big)$$
$$=\frac{n_1-1}{n_0(n_1+n_2)}E_{H_0}\big[1/2-F_1(Y_0)\big]^2+\frac{n_2}{n_0(n_1+n_2)}E_{H_0}\big[1/2-F_1(Y_0)\big]\big[1/2-F_2(Y_0)\big]$$
$$+\frac{n_0-1}{n_0(n_1+n_2)}E_{H_0}\big[F_0(Y_1)-1/2\big]^2-\frac{1}{n_1+n_2}E_{H_0}\big[1/2-F_2(Y_1)\big]\big[F_0(Y_1)-1/2\big]$$
$$-\frac{1}{n_1+n_2}E_{H_0}\big[F_1(Y_2)-1/2\big]\big[F_0(Y_2)-1/2\big]+\frac{1}{4n_0(n_1+n_2)}.$$

Then, the estimator of $\mathrm{cov}_{H_0}\big(\hat{f}_{01}-\hat{f}_{12},\hat{f}_D-1/2\big)$ is given by

$$\widehat{\mathrm{cov}}_{H_0}\big(\hat{f}_{01}-\hat{f}_{12},\hat{f}_D-1/2\big)$$
$$=\frac{n_1}{n_1+n_2}\tilde{\sigma}_{01}^2+\frac{n_2}{(n_1+n_2)n_0^2}\sum_{i=1}^{n_0}\Big[\frac{1}{n_1}\sum_{j=n_0+1}^{n_0+n_1}I(\tilde{y}_i<\tilde{y}_j)-1/2\Big]\Big[\frac{1}{n_2}\sum_{k=n_0+n_1+1}^{n}I(\tilde{y}_i<\tilde{y}_k)-1/2\Big]$$
$$-\frac{1}{n_1(n_1+n_2)}\sum_{j=n_0+1}^{n_0+n_1}\Big[\frac{1}{n_0}\sum_{i=1}^{n_0}I(\tilde{y}_i<\tilde{y}_j)-1/2\Big]\Big[\frac{1}{n_2}\sum_{k=n_0+n_1+1}^{n}I(\tilde{y}_j<\tilde{y}_k)-1/2\Big]$$
$$-\frac{1}{n_2(n_1+n_2)}\sum_{k=n_0+n_1+1}^{n}\Big[\frac{1}{n_0}\sum_{i=1}^{n_0}I(\tilde{y}_i<\tilde{y}_k)-1/2\Big]\Big[\frac{1}{n_1}\sum_{j=n_0+1}^{n_0+n_1}I(\tilde{y}_j<\tilde{y}_k)-1/2\Big].$$

By now, we obtain the estimates of the correlations ρ_x, $x\in\{R,A,D\}$ as

$$\hat{\rho}_x=\widehat{\mathrm{corr}}_{H_0}\big(Z_1,Z_x\big)=\frac{\widehat{\mathrm{cov}}_{H_0}\big(\hat{f}_{01}-\hat{f}_{12},\hat{f}_x-1/2\big)}{\sqrt{\widehat{\mathrm{var}}_{H_0}\big(\hat{f}_{01}-\hat{f}_{12}\big)\widehat{\mathrm{var}}_{H_0}\big(\hat{f}_x-1/2\big)}},\quad x\in\{R,A,D\}.$$

3. Additional simulation results for the model selection procedure

In this section, we show the performances of the proposed genetic model selection procedure. The simulation settings are the same as those in the main text. We consider the t-distributed error term with 3 degrees of freedom. Table S1-S3 shows the results of $\xi = \Phi^{-1}(0.80)$, $\xi = \Phi^{-1}(0.85)$ and $\xi = \Phi^{-1}(0.95)$, respectively. Comparing the results, we find that the true selection rate (TSR) for choosing the additive model increases as ξ increases. For example, when MAF = 0.30, the TSR for choosing the additive model are 61.15%, 70.41%, 80.60%, and 89.98% for $\xi = \Phi^{-1}(0.80), \Phi^{-1}(0.85), \Phi^{-1}(0.90)$ and $\Phi^{-1}(0.95)$, respectively. However, the TSR for choosing the recessive or dominant model decreases when ξ increases. For example, when MAF = 0.50, the TSR for choosing the dominant model are 92.91%, 88.48%, 83.97%, and 72.85% for $\xi = \Phi^{-1}(0.80), \Phi^{-1}(0.85)$, $\Phi^{-1}(0.90)$ and $\Phi^{-1}(0.95)$ respectively. So there is a trade-off between ξ and the TSR. In this article, we choose $\xi = \Phi^{-1}(0.90)$.

Table S1. The true selection rate (%) of genetic model using Z_1 with $\xi = \Phi^{-1}(0.80)$ when the error term follows tGEV(0,0,5,1). The sample size is $n = 1,500$ and $10,000$ replicates are conducted.

True model	REC			ADD			DOM		
MAF\Selection rate	REC	ADD	DOM	REC	ADD	DOM	REC	ADD	DOM
0.05	38.12	50.44	11.44	21.61	54.75	23.64	10.83	50.54	38.63
0.10	53.02	42.91	4.07	19.16	60.18	20.66	4.57	44.74	50.69
0.15	66.92	31.54	1.54	19.96	60.36	19.68	1.95	32.93	65.12
0.20	77.73	21.59	0.68	20.16	60.14	19.70	0.91	24.20	74.89
0.25	84.70	14.90	0.40	19.33	61.41	19.26	0.44	16.60	82.96
0.30	89.06	10.83	0.11	19.72	59.84	20.44	0.19	11.86	87.95
0.35	91.50	8.45	0.05	19.73	60.30	19.97	0.19	8.75	91.06
0.40	93.08	6.86	0.06	18.83	60.89	20.28	0.14	7.10	92.76
0.45	93.56	6.33	0.11	19.85	61.22	18.93	0.05	6.65	93.30
0.50	93.77	6.15	0.08	19.99	60.00	20.01	0.04	6.08	93.88

Table S2. The true selection rate (%) of genetic model using Z_1 with $\xi = \Phi^{-1}(0.85)$ when the error term follows tGEV(0,0,5,1). The sample size is $n = 1,500$ and $10,000$ replicates are conducted.

True model	REC			ADD			DOM		
MAF\Selection rate	REC	ADD	DOM	REC	ADD	DOM	REC	ADD	DOM
0.05	30.98	60.58	8.44	15.38	66.02	18.60	6.39	61.73	31.88
0.10	45.07	52.12	2.81	14.52	69.96	15.52	3.00	53.85	43.15
0.15	60.64	38.21	1.15	15.27	69.22	15.51	1.19	42.65	56.16
0.20	71.28	28.24	0.48	14.57	70.77	14.66	0.43	30.84	68.73
0.25	79.86	19.90	0.24	14.66	70.64	14.70	0.24	22.49	77.27
0.30	84.57	15.35	0.08	14.18	71.53	14.29	0.07	16.76	83.17
0.35	88.03	11.95	0.02	15.73	69.61	14.66	0.08	13.11	86.81
0.40	90.68	9.27	0.05	14.88	70.27	14.85	0.04	9.85	90.11
0.45	91.10	8.86	0.04	14.00	71.10	14.90	0.04	9.31	90.65
0.50	90.99	9.00	0.01	14.67	70.33	15.00	0.02	8.99	90.99

Table S3. The true selection rate (%) of genetic model using Z_1 with $\xi = \Phi^{-1}(0.95)$ when the error term follows tGEV(0,0,5,1). The sample size is $n = 1,500$ and $10,000$ replicates are conducted.

True model	REC			ADD			DOM		
MAF\Selection rate	REC	ADD	DOM	REC	ADD	DOM	REC	ADD	DOM
0.05	7.29	91.65	1.06	1.67	92.85	5.48	0.18	86.05	13.77
0.10	21.06	78.36	0.58	3.85	90.72	5.43	0.40	77.54	22.06
0.15	33.65	66.24	0.11	4.32	90.68	5.00	0.22	66.50	33.28
0.20	46.71	53.23	0.06	4.28	91.01	4.71	0.04	54.76	45.20
0.25	57.48	42.48	0.04	4.80	90.07	5.13	0.01	44.53	55.46
0.30	65.53	34.46	0.01	5.02	89.98	5.00	0.01	35.73	64.26
0.35	71.29	28.71	0.00	4.59	90.08	5.33	0.01	29.03	70.96
0.40	74.59	25.41	0.00	4.92	90.01	5.07	0.00	25.77	74.23
0.45	76.61	23.39	0.00	5.00	90.27	4.73	0.00	23.70	76.30
0.50	76.40	23.60	0.00	4.99	90.01	5.00	0.00	22.77	77.23

4. Simulation results for the error term following the generalized extreme distribution.

We compare the performances of four procedures: KW, Z_A, MAX3, and TPP. Here, we consider the linear model $Y = \beta_0 + G\beta_1 + \epsilon$, where Y denotes the phenotype value, G denotes the genotype value, and the error term ϵ follows a generalized extreme value distribution (a heavy-tailed distribution) with the shape parameter 0, the location parameter 0, and the scale parameter d (denoted as GEV(0, 0, d)).

Table S4 shows the adjusted α^* of the TPP under the null hypothesis when the error term follows a generalized extreme value distribution. When the nominal level is 0.05, we calculate the mean and standard deviation (SD) with $d = 2$ based on 1,000 replicates. 50,000 replicates are conducted for the nominal level of 0.001 with $d = 1$. The results are similar to those of the t-distributed error term and show that the adjusted level is always less than the nominal significant level. When $\alpha = 0.05, d = 2$, and MAF=0.15, the adjusted level α^* is 0.0328. Also, the standard deviations of the adjusted levels can be omitted compared to their means.

Table S5 shows the empirical type I errors of the four tests under the significant level of 0.05 and 0.001. The sample size is 1,000. Here we use $\xi = \Phi^{-1}(0.90)$, $\beta_0 = 0.5$, $d \in \{1, 2\}$, and $p \in \{0.05, 0.1, \cdots, 0.5\}$. 1,000 replicates are conducted for the nominal significant level of 0.05 with $d = 2$ and 50,000 replicates are conducted for the nominal significant level of 0.001 with $d = 1$. The results show that all of the four tests could control the type I error correctly with the empirical values being close to the nominal significance levels. For example, when MAF=0.40 and the nominal level is 0.05, the empirical type I error rates for KW, Z_A, MAX3, and TPP are 0.049, 0.055, 0.045, and 0.044, respectively.

Figure S1 reports the power results of KW, Z_A, MAX3 and TPP under the recessive,

additive, and dominant model. To make the power comparable, we set $\beta_1 = \ln 1.2, d = 2$ for the nominal level of 0.05 and $\beta_1 = \ln 1.2, d = 1$ for the nominal level of 0.001. Based on the results, our proposed TPP is more powerful than KW, Z_A and MAX3 under most of the considered scenarios. For example, when the genetic model is recessive and MAF=0.40, the powers of KW, Z_A, MAX3, and TPP under the nominal level of 0.05 are 0.352, 0.277, 0.367, and 0.394, respectively. Furthermore, the TPP has greater robustness than KW, Z_A and MAX3.

Table S4. The adjusted level α^* for the nominal level $\alpha = 0.05$ and 0.001 when the error term follows a GEV distribution. 1,000 replicates are for the nominal level 0.05 and 50,000 replicates are for the level 0.001.

	MAF	0.05	0.10	0.15	0.20	0.25	0.30	0.35	0.40	0.45	0.50
$\alpha = 0.05$	mean	0.0382	0.0338	0.0328	0.0319	0.0312	0.0305	0.0300	0.0296	0.0294	0.0293
	sd	0.00802	0.00183	0.00151	0.00122	0.00102	0.00083	0.00072	0.00069	0.00068	0.00067
$\alpha = 0.05$	mean	0.00074	0.00064	0.00063	0.00061	0.00059	0.00058	0.00057	0.00056	0.00056	0.00056
	sd	0.000161	0.000046	0.000031	0.000028	0.000019	0.000013	0.000008	0.000005	0.000003	0.000002

Table S5. The empirical type I errors of KW, Z_A, MAX3, and TPP when the error term follows a GEV distribution. The total number of the subjects is $n = 1,000$. The left panel is for the significant level $\alpha = 0.05$ and d=2 and the right panel is for the significant level $\alpha = 0.001$ and $d = 1$.

	$\alpha = 0.05$				$\alpha = 0.001$			
MAF	KW	Z_A	MAX3	TPP	KW	Z_A	MAX3	TPP
0.05	0.041	0.065	0.030	0.048	0.00058	0.00092	0.00058	0.00074
0.10	0.042	0.058	0.038	0.043	0.00074	0.00080	0.00052	0.00048
0.15	0.048	0.051	0.047	0.047	0.00134	0.00080	0.00072	0.00064
0.20	0.043	0.045	0.035	0.032	0.00076	0.00050	0.00054	0.00060
0.25	0.064	0.041	0.061	0.052	0.00102	0.00090	0.00090	0.00080
0.30	0.063	0.052	0.057	0.050	0.00084	0.00072	0.00068	0.00054
0.35	0.037	0.040	0.036	0.030	0.00078	0.00086	0.00074	0.00070
0.40	0.049	0.055	0.045	0.044	0.00106	0.00100	0.00108	0.00082
0.45	0.046	0.042	0.040	0.031	0.00106	0.00084	0.00088	0.00060
0.50	0.053	0.056	0.055	0.035	0.00102	0.00086	0.00110	0.00080

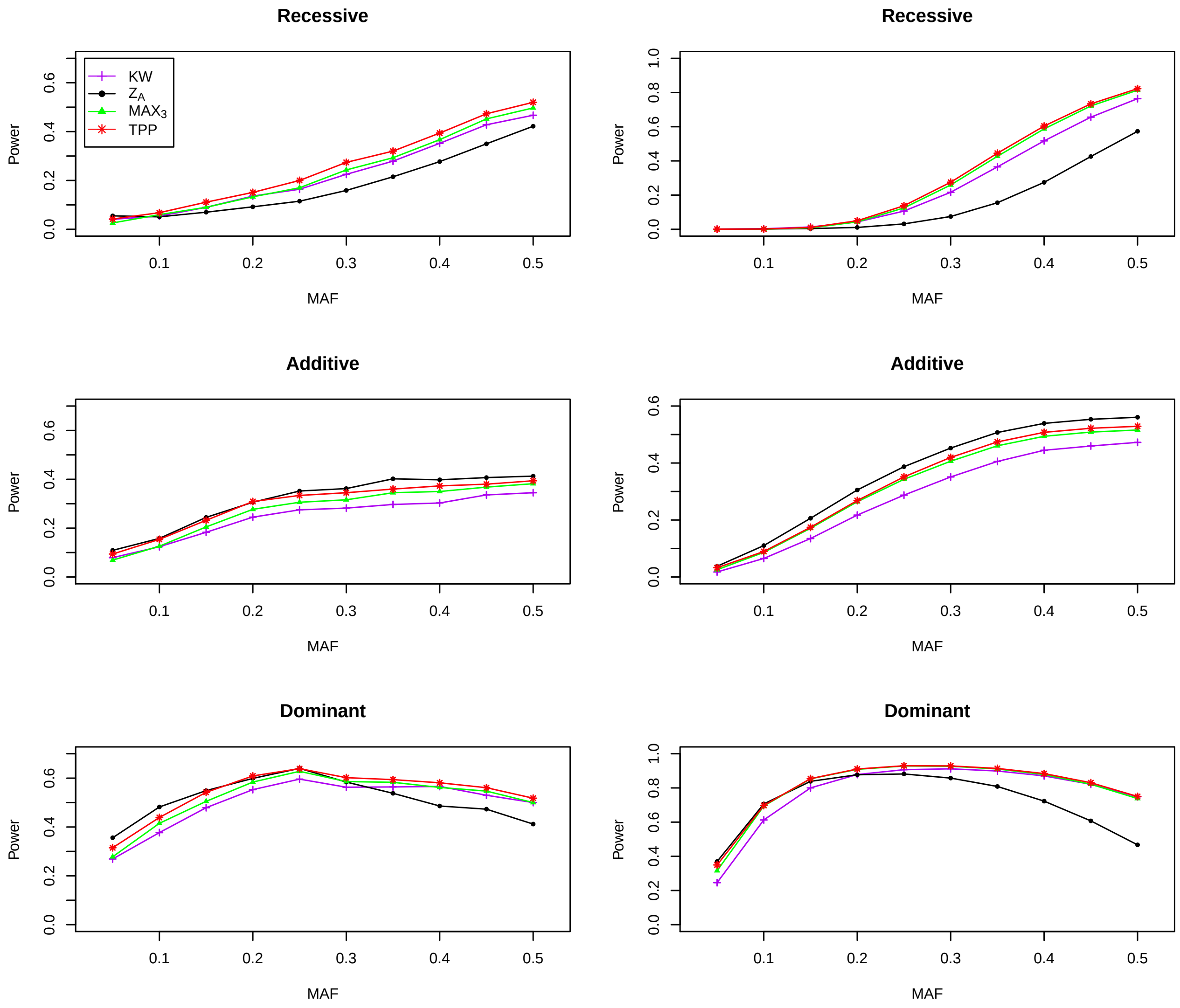

Figure S1. The powers of KW, Z_A, MAX3, and TPP with the GEV-distributed error term under three different models. The first column is for the nominal level $\alpha = 0.05$ and The second column is for the nominal level $\alpha = 0.001$. The total number of the subjects is $n = 1,000$.

5. Simulation results for the error term following the centralized t distribution.

We compare the performances of four procedures: KW, Z_A, MAX3, and TPP. Here, we consider the linear model $Y = \beta_0 + G\beta_1 + \epsilon$, where Y denotes the phenotype value, G denotes the genotype value, and the error term ϵ follows a centralized t distribution with b degrees of freedom (denoted as $t(b)$). Let $b = 3$ and the sample size is 1,000.

Table S6 shows the adjusted α^* of the TPP under the null hypothesis when the error term follows a centralized t distribution. When the nominal level is 0.05, we calculate the mean and standard deviation (SD) with $b = 3$ based on 1,000 replicates. 50,000 replicates are conducted for the nominal level of 0.001 with $b = 3$. The results are similar to those of the GEV-distributed error term and show that the adjusted level is always less than the nominal significant level. When $\alpha = 0.05$, and MAF=0.15, the adjusted level α^* is 0.0356. Also, the standard deviations of the adjusted levels can be omitted compared to their means.

Table S7 shows the empirical type I errors of the four tests under the significant level of 0.05 and 0.001. Here we use $\xi = \Phi^{-1}(0.90)$, $\beta_0 = 0.5$, and $p \in \{0.05, 0.1, \cdots, 0.5\}$. 2,000 replicates are conducted for the nominal significant level of 0.05 and 50,000 replicates are conducted for the nominal significant level of 0.001. The results show that all of the four tests could control the type I error correctly with the empirical values being close to the nominal significance levels. For example, when MAF=0.40 and the nominal level is 0.05, the empirical type I error rates for KW, Z_A, MAX3, and TPP are 0.055, 0.043, 0.054 and 0.041, respectively.

Figure S2 reports the power results of KW, Z_A, MAX3 and TPP under the recessive, additive, and dominant model. To make the power comparable, we set $\beta_1 = \ln 1.2$ for the

nominal level of 0.05 and 0.001. Based on the results, our proposed TPP is more powerful than KW, Z_A and MAX3 under most of the considered scenarios. For example, when the genetic model is recessive and MAF=0.50, the powers of KW, Z_A, MAX3, and TPP for the nominal level of 0.001 are 0.639, 0.409, 0.658, and 0.684 respectively. Furthermore, the TPP has greater robustness than KW, Z_A and MAX3.

Table S6. The adjusted level α^* with the nominal significant level α of 0.05 and 0.001 for a centralized t-distribution error. 1,000 replicates are for the nominal level 0.05 and 50,000 replicates are for the level 0.001.

	MAF	0.05	0.10	0.15	0.20	0.25	0.30	0.35	0.40	0.45	0.50
$\alpha = 0.05$	mean	0.0378	0.0356	0.0356	0.0358	0.0360	0.0362	0.0364	0.0365	0.0366	0.0367
	sd	0.00536	0.00064	0.00036	0.00031	0.00029	0.00028	0.00028	0.00027	0.00029	0.00028
$\alpha = 0.001$	mean	0.00075	0.00069	0.00067	0.00066	0.00065	0.00064	0.00064	0.00063	0.00063	0.00063
	sd	0.000158	0.000028	0.000020	0.000017	0.000013	0.000009	0.000007	0.000005	0.000005	0.000004

Table S7. The empirical type I errors of KW, Z_A, MAX3, and TPP when the error term follows the t-distribution with 3 degrees of freedom. The sample size is 1,000. The left panel is for the significant level $\alpha = 0.05$ and the right panel is for the significant level $\alpha = 0.001$.

	$\alpha = 0.05$				$\alpha = 0.001$			
MAF	KW	Z_A	MAX3	TPP	KW	Z_A	MAX3	TPP
0.05	0.041	0.047	0.023	0.032	0.00052	0.00068	0.00038	0.00050
0.10	0.043	0.052	0.042	0.042	0.00060	0.00074	0.00046	0.00048
0.15	0.052	0.053	0.047	0.044	0.00098	0.00082	0.00076	0.00082
0.20	0.043	0.054	0.043	0.053	0.00096	0.00078	0.00094	0.00072
0.25	0.043	0.046	0.046	0.047	0.00112	0.00086	0.00088	0.00076
0.30	0.059	0.043	0.052	0.041	0.00108	0.00076	0.00084	0.00078
0.35	0.048	0.054	0.041	0.049	0.00112	0.00086	0.00098	0.00092
0.40	0.055	0.043	0.054	0.041	0.00086	0.00074	0.00094	0.00074
0.45	0.039	0.037	0.042	0.037	0.00116	0.00090	0.00102	0.00074
0.50	0.039	0.043	0.032	0.044	0.00098	0.00082	0.00104	0.00086

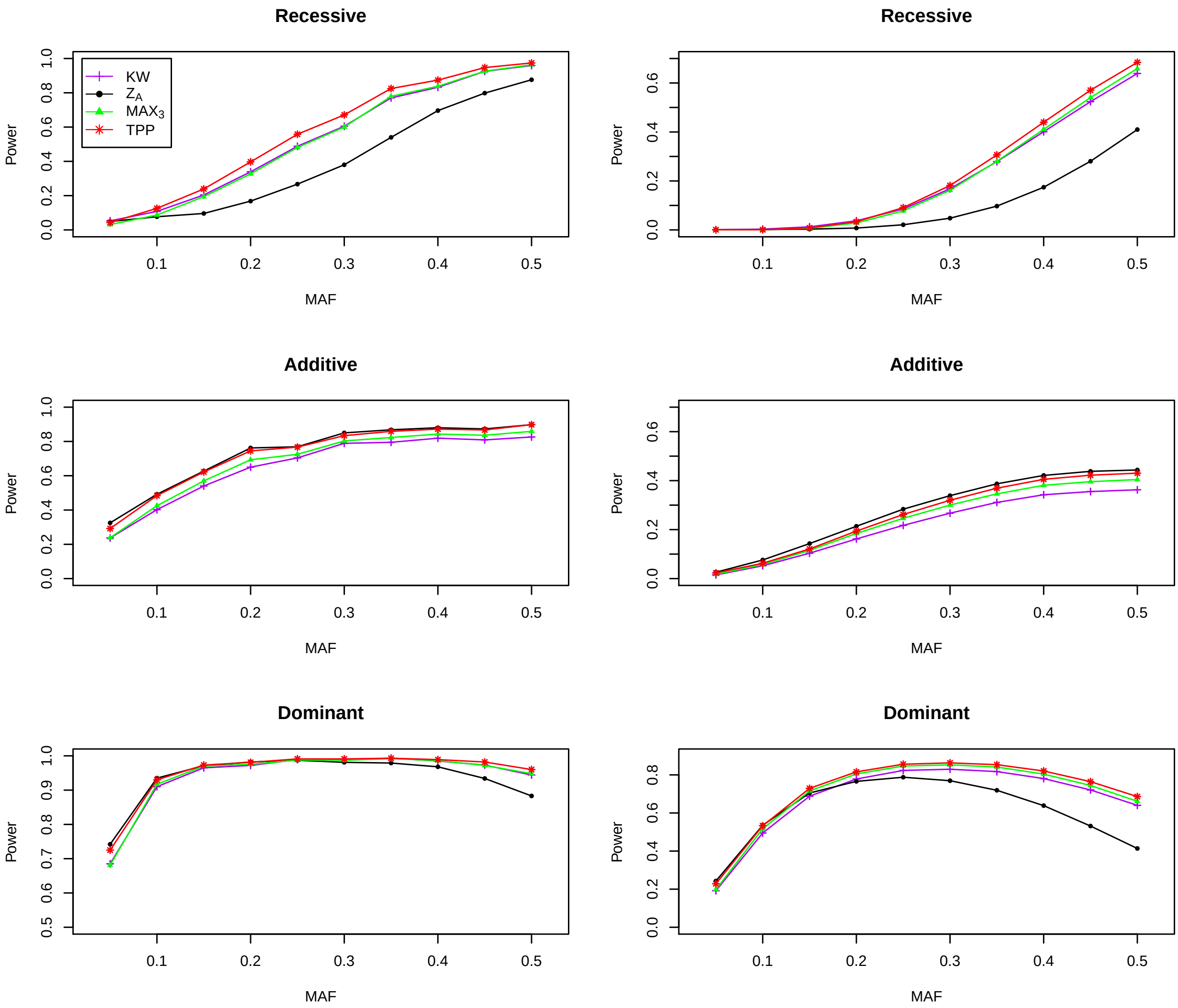

Figure S2. The powers of KW, Z_A, MAX3, and TPP with the t-distribution error under three genetic models. The first column is for the nominal level $\alpha = 0.05$ and the second column is for the nominal level $\alpha = 0.001$. The total number of the subjects is $n = 1,000$.

6. Simulation results for the model with covariates.

Here, we simulated the data from the linear model with covariates: $Y = \beta_0 + X\gamma + G\beta_1 + \epsilon$, where Y denotes the phenotype value, G denotes the genotype value at a SNP locus, X denotes the covariate value which follows a standard normal distribution, and ϵ follows a truncated generalized extreme value distribution (a heavy-tailed distribution, denoted as tGEV(0, 0, d, 0)) with the shape parameter 0, the location parameter 0, the scale parameter d, and the truncated point 0. We set $\beta_0 = 0.5, \gamma = 0.5, \beta_1 = \{0.25, 0.50\}$, $d = 5$, and the MAF $p \in \{0.05, 0.1, \cdots, 0.5\}$. The total sample size is 1,500. And we still use $\xi = \Phi^{-1}(0.90)$.

Table S8 shows the adjusted α^* of the TPP under the null hypothesis. The parameters are the same as above. For the nominal level of 0.05 and 0.001, we calculate the means and standard deviations of the adjusted significant level based on 2,000 and 50,000 replicates, respectively. The results are similar to those of the model without considering covariates. Similarly, the adjusted level is always less than the nominal significant level α. And the value of α^* is relatively stable because its standard deviations can be omitted compared with the means. For example, when MAF=0.30, the adjusted levels for the nominal level $\alpha = 0.05$ and $\alpha = 0.001$ are 0.0303 and 0.00058, respectively, and the corresponding SD are 0.00093 and 0.000015, respectively.

The results of the empirical type I error rates of five tests: KW, Z_R, Z_A, MAX3, and TPP are presented in Table S9. Here we set $\xi = \Phi^{-1}(0.90)$, $\beta_0 = 0.5$, $\gamma = 0.5$, and $p \in \{0.05, 0.1, \cdots, 0.5\}$. The sample size is 1,500. 2,000 replicates are conducted for the nominal significant level of 0.05 and 50,000 replicates are conducted for the nominal significant level of 0.001. We still consider two nominal level of 0.05 and 0.001. The results in Table S9 show that all of the five tests could control the type I error correctly

with the empirical values being close to the nominal significance level. For example, when MAF=0.35, the empirical type I error rates of KW, Z_R, Z_A, MAX3, and TPP test are 0.048, 0.051, 0.052, 0.052, and 0.044, respectively, under the significant level of 0.05.

We compare the performance of power among five tests: KW, Z_R, Z_A, MAX3 and TPP and the results for the nominal level of 0.05 and 0.001 are showed in Figure S3 and Figure S4, respectively. In order to make the power comparable, when the nominal level is 0.001, we specify $d = 3$ for $\beta_1 = 0.25$ and $d = 5$ for $\beta_1 = 0.50$. we set $d = 5$ and $\beta_1 = \{0.25, 0.50\}$ for the nominal level of 0.05. The comparison results are similar to those of the model without covariates. For most scenarios, the proposed TPP is superior in power than the other tests. Specifically, TPP is more powerful than KW, Z_A and MAX3 under the recessive model and when the true genetic model is additive, TPP performs better than KW, Z_R and MAX3. For example, when MAF is 0.20, $\beta_1 = 0.50$, $\alpha = 0.05$ and the genetic model is recessive, the empirical powers of KW, Z_R, Z_A, MAX3, and TPP are 0.358, 0.523, 0.192, 0.433, and 0.483, respectively. When MAF is 0.40, $\beta_1 = 0.25$, $\alpha = 0.05$, and the genetic model is dominant, the empirical powers of KW, Z_R, Z_A, MAX3, and TPP are 0.513, 0.108, 0.488, 0.528, and 0.545, respectively. In addition, TPP is more robust against the genetic model than the other four tests. For example, when $\alpha = 0.05$ and $\beta_1 = 0.50$, the minimum value of power for TPP over MAF from 0.10 to 0.50 is 0.142, which is larger than those of KW (0.099), Z_R (0.097), Z_A (0.068), and MAX3 (0.116).

Table S8. The adjusted level α^* with the nominal significant level α of 0.05 and 0.001 for the model with covaraites. The error term follows tGEV(0,0,5,0). 2,000 replicates are for the nominal level 0.05 and 50,000 replicates are for the level 0.001.

	MAF	0.05	0.10	0.15	0.20	0.25	0.30	0.35	0.40	0.45	0.50
$\alpha = 0.05$	mean	0.0366	0.0336	0.0327	0.0319	0.0310	0.0303	0.0297	0.0293	0.0291	0.0290
	sd	0.00709	0.00172	0.00173	0.00152	0.00122	0.00093	0.00074	0.00063	0.00058	0.00056
$\alpha = 0.001$	mean	0.00071	0.00063	0.00063	0.00061	0.00059	0.00058	0.00057	0.00056	0.00056	0.00056
	sd	0.000152	0.000038	0.000033	0.000032	0.000023	0.000015	0.000009	0.000005	0.000003	0.000002

Table S9. The empirical type I errors of KW, Z_R, Z_A, MAX3, and TPP for the model with covariates. The error term follows tGEV(0,0,5,0). The sample size is 1,500. The left panel is for the significant level $\alpha = 0.05$ and the right panel is for the significant level $\alpha = 0.001$.

	$\alpha = 0.05$					$\alpha = 0.001$				
MAF	KW	Z_R	Z_A	MAX3	TPP	KW	Z_R	Z_A	MAX3	TPP
0.05	0.047	0.029	0.055	0.033	0.046	0.00058	0.00030	0.00082	0.00060	0.00062
0.10	0.041	0.045	0.045	0.039	0.039	0.00078	0.00044	0.00096	0.00054	0.00060
0.15	0.043	0.044	0.043	0.043	0.044	0.00106	0.00054	0.00108	0.00100	0.00080
0.20	0.051	0.052	0.047	0.049	0.040	0.00098	0.00082	0.00080	0.00094	0.00074
0.25	0.051	0.051	0.050	0.047	0.039	0.00092	0.00086	0.00084	0.00092	0.00082
0.30	0.046	0.047	0.045	0.047	0.042	0.00108	0.00090	0.00072	0.00118	0.00088
0.35	0.048	0.051	0.052	0.052	0.044	0.00078	0.00068	0.00050	0.00060	0.00036
0.40	0.053	0.048	0.050	0.047	0.040	0.00078	0.00094	0.00066	0.00094	0.00084
0.45	0.047	0.050	0.047	0.051	0.044	0.00088	0.00076	0.00070	0.00098	0.00082
0.50	0.049	0.044	0.048	0.050	0.038	0.00070	0.00072	0.00078	0.00098	0.00080

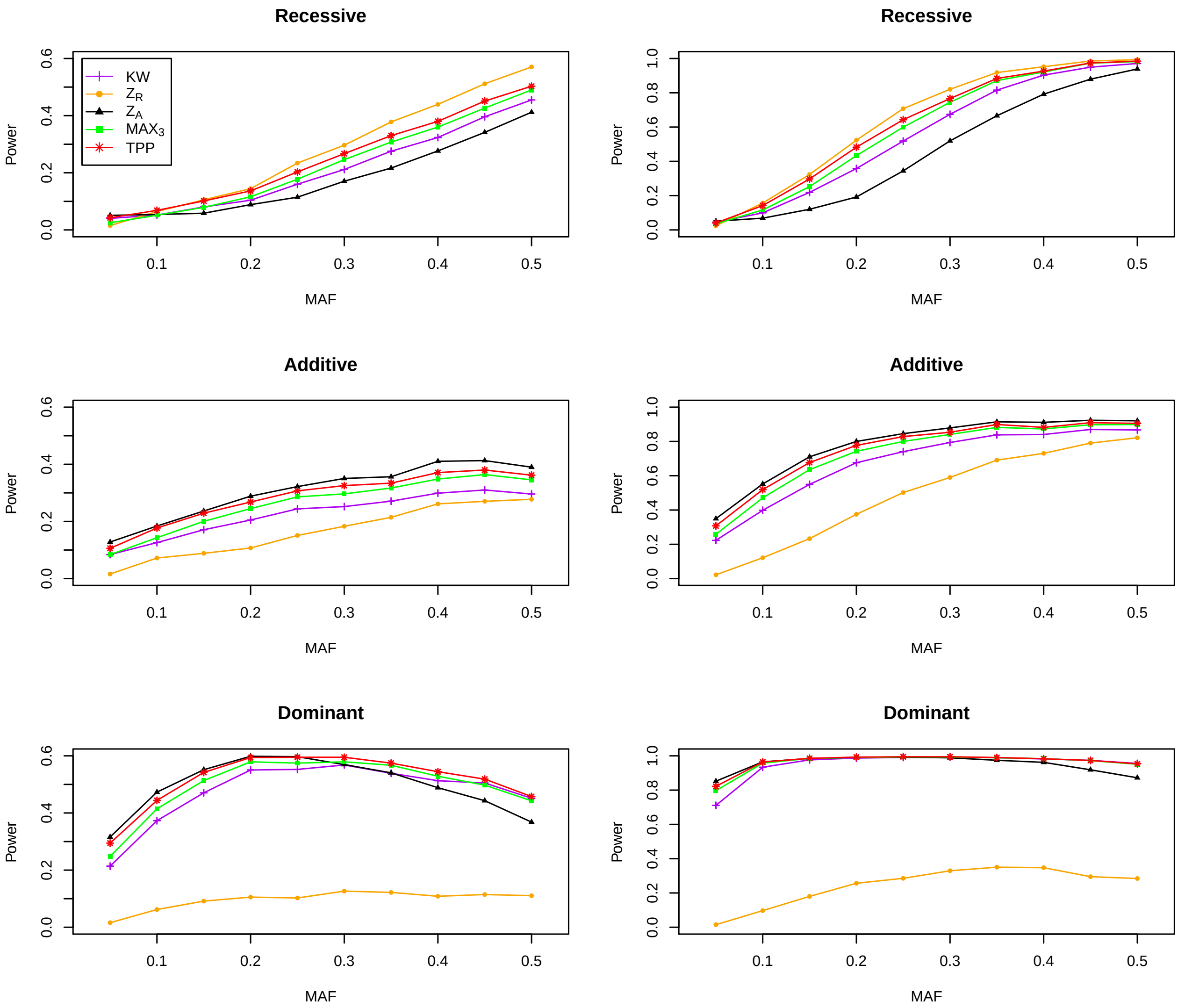

Figure S3. The powers of KW, Z_R, Z_A, MAX3, and TPP for the model with covariates under three genetic models. The error term follows tGEV(0,0,d,0). The nominal level is 0.05. The first column is for $\beta_1 = 0.25$ and $d = 5$. The second column is for $\beta_1 = 0.50$ and $d = 5$.

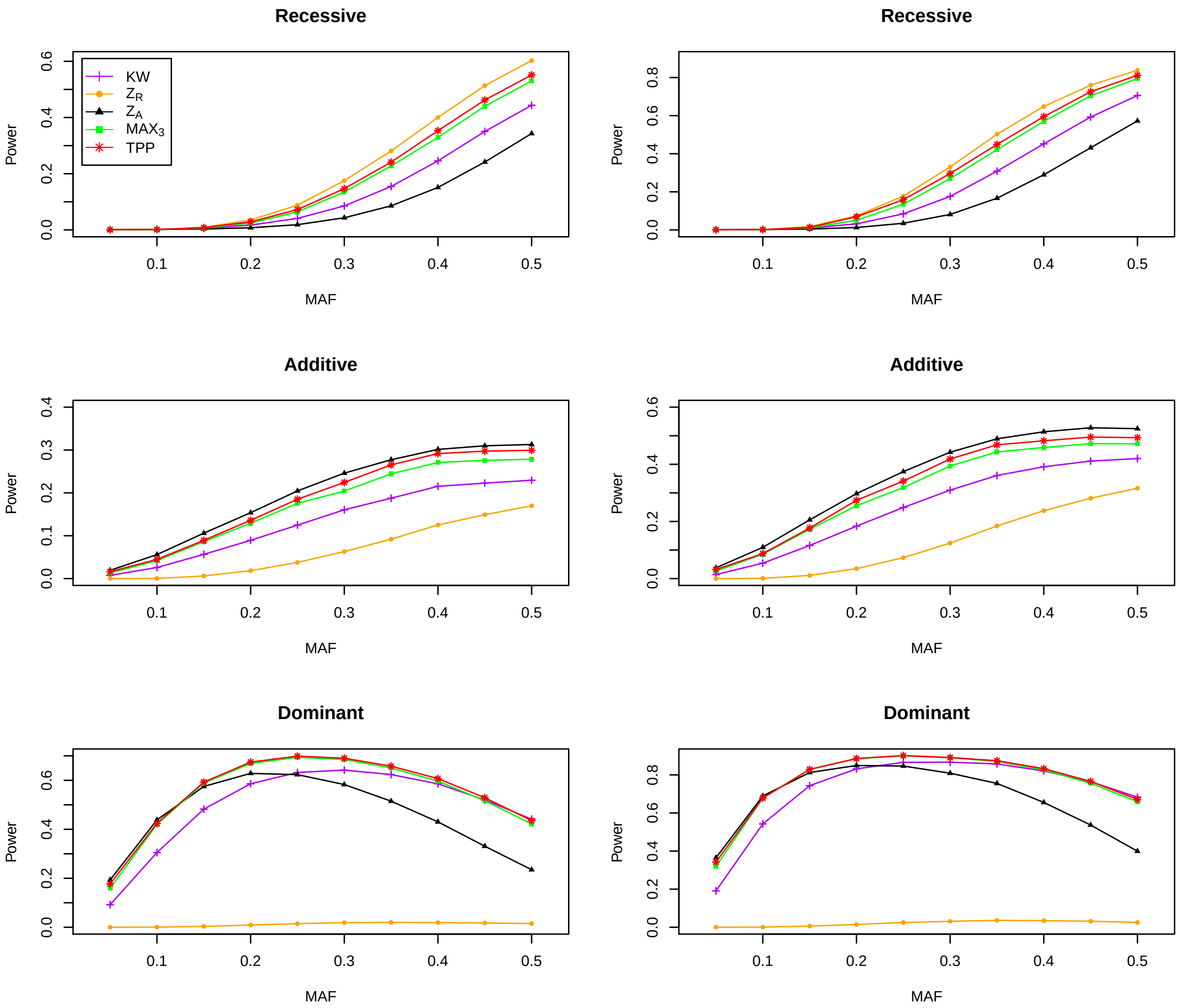

Figure S4. The powers of KW, Z_R, Z_A, MAX3, and TPP for the model with covariates under three genetic models. The error term follows tGEV(0,0,d,0). The nominal level is 0.001. The first column is for $\beta_1 = 0.25$ and $d = 3$. The second column is for $\beta_1 = 0.50$ and $d = 5$.

7. Additional p-value results of the SNPs in gene *DNAH9* for the association with Anti-CCP Measure.

Table S10. The p-values of the other 69 SNPs in gene *DNAH9* for the association with Anti-CCP Measure. α^* is the adjusted p-value threshold for 5×10^{-5}.

snpid	KW	Z_A	MAX3	TPP	Genetic model	α^*
rs2079719	0.8259	0.9375	0.8231	0.9375	ADD	3.04×10^{-5}
rs4792152	0.4716	0.3393	0.4020	0.3393	ADD	2.93×10^{-5}
rs929466	0.6514	0.3451	0.5720	0.3451	ADD	2.86×10^{-5}
rs758424	0.3946	0.1556	0.2938	0.1556	ADD	2.87×10^{-5}
rs12936449	0.4726	0.2500	0.4395	0.2500	ADD	3.12×10^{-5}
rs9912519	0.1602	0.1629	0.2118	0.1629	ADD	3.15×10^{-5}
rs11649836	0.1415	0.1783	0.1090	0.4128	REC	3.27×10^{-5}
rs1010797	0.5810	0.6150	0.5295	0.6150	ADD	2.94×10^{-5}
rs8078427	0.3985	0.9255	0.4977	0.2904	DOM	2.92×10^{-5}
rs3744575	0.4519	0.2573	0.4484	0.2573	ADD	3.40×10^{-5}
rs10521184	0.7113	0.6078	0.6772	0.6078	ADD	2.97×10^{-5}
rs11658293	0.2212	0.0977	0.1755	0.0977	ADD	2.92×10^{-5}
rs4792159	0.2270	0.0944	0.1858	0.0944	ADD	2.91×10^{-5}
rs3744576	0.2175	0.0852	0.1690	0.0852	ADD	2.91×10^{-5}
rs17601333	0.8672	0.7252	0.9033	0.7252	ADD	3.57×10^{-5}
rs2108961	0.3163	0.6721	0.4086	0.6721	ADD	2.77×10^{-5}
rs7221991	0.9769	0.8546	0.6287	0.8546	ADD	7.97×10^{-5}

(continued)

Table S10., *continued*

snpid	KW	Z_A	MAX3	TPP	Genetic model	α^*
rs2322047	0.3851	0.1662	0.3162	0.1662	ADD	7.96×10^{-5}
rs11651009	0.6809	0.6975	0.6560	0.6975	ADD	3.03×10^{-5}
rs8081897	0.0698	0.0695	0.1356	0.0695	ADD	3.26×10^{-5}
rs12948671	0.2090	0.1227	0.1542	0.1227	ADD	2.97×10^{-5}
rs8074402	0.8314	0.6248	0.8159	0.6248	ADD	3.59×10^{-5}
rs8073778	0.5499	0.5499	0.4983	0.5499	ADD	3.57×10^{-5}
rs9898602	0.0880	0.0297	0.0608	0.3223	DOM	7.93×10^{-5}
rs3744581	0.4698	0.3873	0.4644	0.3873	ADD	2.98×10^{-5}
rs12449476	0.1956	0.0798	0.1560	0.0798	ADD	3.12×10^{-5}
rs3744583	0.0072	0.0045	0.0042	0.0980	DOM	2.89×10^{-5}
rs11078030	0.0750	0.0808	0.1583	0.0925	DOM	5.13×10^{-5}
rs11078031	0.0160	0.0169	0.0079	0.2524	DOM	2.87×10^{-5}
rs12603082	0.4507	0.2321	0.4142	0.2321	ADD	3.53×10^{-5}
rs4539641	0.8778	0.6192	0.8436	0.6192	ADD	3.17×10^{-5}
rs7225157	0.1663	0.0655	0.1317	0.0655	ADD	3.56×10^{-5}
rs11658421	0.7072	0.8970	0.7289	0.8970	ADD	3.66×10^{-5}
rs2158971	0.6936	0.4564	0.6347	0.4564	ADD	2.91×10^{-5}
rs4792176	0.9447	0.9909	0.9716	0.9909	ADD	2.87×10^{-5}
rs2010253	0.7613	0.4881	0.7351	0.4881	ADD	2.94×10^{-5}
rs7225975	0.6512	0.4134	0.6028	0.4134	ADD	3.19×10^{-5}
rs2322052	0.3083	0.6094	0.3193	0.1720	DOM	2.88×10^{-5}
rs9894590	0.2431	0.0932	0.1832	0.0932	ADD	2.95×10^{-5}

(continued)

Table S10., *continued*

snpid	KW	Z_A	MAX3	TPP	Genetic model	α^*
rs12949783	0.9521	0.7816	0.9381	0.7816	ADD	2.86×10^{-5}
rs4792181	0.7908	0.5590	0.7558	0.5590	ADD	2.91×10^{-5}
rs717788	0.3543	0.2077	0.3006	0.2077	ADD	2.88×10^{-5}
rs9892829	0.4652	0.2297	0.4088	0.2297	ADD	2.88×10^{-5}
rs11078034	0.8250	0.8958	0.8750	0.8958	ADD	2.91×10^{-5}
rs8182254	0.3810	0.2161	0.3285	0.2161	ADD	2.88×10^{-5}
rs12946763	0.7251	0.6764	0.7986	0.6764	ADD	3.19×10^{-5}
rs4548916	0.3546	0.1521	0.2836	0.1521	ADD	2.90×10^{-5}
rs11869944	0.8030	0.5406	0.7652	0.5406	ADD	2.89×10^{-5}
rs11651928	0.5704	0.8589	0.7145	0.8589	ADD	2.86×10^{-5}
rs7219827	0.5384	0.7008	0.5698	0.7008	ADD	2.86×10^{-5}
rs8070243	0.7854	0.4883	0.7357	0.4883	ADD	2.88×10^{-5}
rs12452241	0.0230	0.7197	0.1336	0.0687	REC	3.05×10^{-5}
rs4792189	0.9279	0.7528	0.9314	0.7528	ADD	3.36×10^{-5}
rs9902986	0.7207	0.4424	0.6855	0.4424	ADD	2.88×10^{-5}
rs2240519	0.7061	0.4642	0.6476	0.4642	ADD	2.87×10^{-5}
rs2041072	0.4182	0.3611	0.4962	0.3611	ADD	3.47×10^{-5}
rs4792192	0.4354	0.4084	0.4955	0.4084	ADD	3.44×10^{-5}
rs3815269	0.4126	0.3530	0.4956	0.3530	ADD	3.43×10^{-5}
rs9896353	0.2231	0.6806	0.4344	0.2498	DOM	3.17×10^{-5}
rs12453561	0.3936	0.1669	0.3116	0.1669	ADD	3.13×10^{-5}

(continued)

Table S10., *continued*

snpid	KW	Z_A	MAX3	TPP	Genetic model	α^*
rs12453581	0.1975	0.6697	0.4162	0.4381	DOM	3.10×10^{-5}
rs3785944	0.6095	0.3800	0.5310	0.3800	ADD	2.98×10^{-5}
rs12940988	0.5403	0.3106	0.4705	0.3106	ADD	2.99×10^{-5}
rs12940825	0.6413	0.7256	0.6633	0.7256	ADD	2.86×10^{-5}
rs11651333	0.7272	0.7954	0.7550	0.7954	ADD	2.87×10^{-5}
rs2190616	0.7672	0.6472	0.7335	0.6472	ADD	2.87×10^{-5}
rs12449769	0.3653	0.1701	0.3158	0.1701	ADD	3.06×10^{-5}
rs12452927	0.7069	0.6941	0.7004	0.6941	ADD	2.87×10^{-5}
rs2322140	0.7222	0.6062	0.6843	0.6062	ADD	2.87×10^{-5}
